# Genomic landscape analyses of reprogrammed cells using integrative and non-integrative methods reveal variable cancer-associated alterations

**DOI:** 10.18632/oncotarget.26857

**Published:** 2019-04-12

**Authors:** Frank Griscelli, Christophe Desterke, Olivier Feraud, Dominique Divers, Noufissa Oudrhiri, Lucie Tosca, Ali G. Turhan, Annelise Bennaceur-Griscelli

**Affiliations:** ^1^ Institut National de la Santé et de la Recherche Médicale (INSERM) U935, Paris, France; ^2^ Human Embryonic Stem Cell Core Facility, ESTeam Paris Sud-INGESTEM, Villejuif, France; ^3^ Université Paris Descartes, Sorbonne Paris Cité, Faculté des Sciences Pharmaceutiques et Biologiques, Paris, France; ^4^ Institut Gustave-Roussy, Département de Biologie et de Pathologie Médicales, Villejuif, France; ^5^ Université Paris-Sud, Faculté de Médecine Kremlin Bicêtre, Le Kremlin-Bicêtre, France; ^6^ Service d’Hématologie Biologique, Hôpital Universitaire Paris Sud, (AP-HP) Kremlin Bicêtre, Le Kremlin-Bicêtre, France

**Keywords:** iPSCs, genetic instability, CGH array

## Abstract

Recent development of cell reprogramming technologies brought a major hope for future cell therapy applications by the use of these cells or their derivatives. For this purpose, one of the major requirements is the absence of genomic alterations generating a risk of cell transformation. Here we analyzed by microarray-based comparative genomic hybridization human iPSC generated by two non-integrative and one integrative method at pluripotent stage as well as in corresponding teratomas. We show that all iPSC lines exhibit copy number variations (CNV) of several genes deregulated in oncogenesis. These cancer-associated genomic alterations were more pronounced in virally programmed hiPSCs and their derivative teratoma as compared to those found in iPSC generated by mRNA-mediated reprogramming. Bioinformatics analysis showed the involvement of these genes in human leukemia and carcinoma. We conclude that genetic screening should become a standard procedure to ensure that hiPSCs are free from cancer-associated genomic alterations before clinical use.

## INTRODUCTION

Random genomic alterations are frequently observed in human induced pluripotent stem cells (hiPSCs) during cell reprogramming essentially due to the massive genome remodeling and it is therefore of major interest to evaluate the genomic status of the cells for clinical use in order to determine that they are free from cancer-associated genomic alterations. Several studies have evaluated the Copy Number Variation (CNV) rates by microarray-based comparative genomic hybridization analysis (aCGH) in hiPSCs during the reprogramming process, showing that non-integrating methods result in fewer *de novo* genomic rearrangements compared with integrative methods [1, 2]. It has also shown that the CNV generated during the early phase of hiPSCs establishment can induce a growth or survival disadvantage which generates genetic mosaicism with the selection during the passage of hiPSCs colonies with less damaged cells [1]. However, this negative selection does not exclude the possibility that during the early phase some minor hazardous genomic alterations, undetectable by aCGH, can confer a survival advantage to a small contingent of cells, which can rapidly take over a genomically normal cell population over time. This will be revealed by aCGH only in cells undergoing long-term differentiation. For this issue a teratoma model will represent a highly selective method allowing revelation by selective pressure, a small subpopulation of cells with a tumor phenotype which can rapidly take over a population undergoing a normal differentiation.

In this study, we assessed cancer-associated genomic alterations by aCGH analysis in hiPSC lines generated by integrative and non-integrative strategies. We have used hiPSC generated by lentiviral mediated pluripotency gene transfer as a category of hiPSC with high risk of cancer whereas in the second category we have analyzed hiPSCgenerated by Sendaï-virus-mediated [3] and mRNA-mediated [4] reprogramming strategies. We compared these three categories of hiPSC by using “PluriNet network”, previously shown to be an efficient tool to define protein-protein network shared by pluripotent stem cells (hESC and hiPSCs) and to be a useful biologically inspired gauge for classifying pluripotent stem cells phenotypes [5]. We then assessed the CNV rates matching with “catalogue of somatic mutations in cancer” (COSMIC) database and gene loci involved in human cancer development [6] which appeared *de novo* in both undifferentiated hiPSCs and corresponding teratoma. The analysis of these experiments show that either lentiviral or Sendaï-virus mediated reprogramming is associated with significantly higher numbers of tumorigenic CNVs in both hiPSCs and in teratoma as compared to hiPSC generated with mRNA-mediated pluripotency gene transfer.

## RESULTS

### Analysis of genomic integrity by CGH array of hiPSCs produced by three different reprogramming strategies

The CNV were analyzed using microarray-based comparative genomic hybridization (array-CGH 12x135K Whole-Genome Tiling v3.0) on hiPSCs produced by lentiviral (*n* = 6, passage 14 ± 4) Sendai (*n* = 3, passage 15 ± 2) or mRNA transductions (*n* = 3, passage 16 ± 1) by excluding polymorphic variants described in Toronto Database of Genomic Variants (http://projects.tcag.ca/cgi-bin/variation/gbrowse/hg19) and the CNV observed in parental cells permitting to determine only the CNV that appeared *de novo* during the reprogramming process (Supplementary Figure 1). The residual transgene expression in the lentiviral iPS lines and the elimination of the Sendai virus RNA in the Sendai-derived lines were evaluated by qRT-PCR in iPSCs that were collected at different passages. The study results revealed that all iPSCs produced by the lentiviral method and analysis by CGH arrays still expressed one or two transcriptional factors (OSLN) between 10 and 14 passages and a clearance of the vectors was observed only after 20 to 32 passages (Supplementary Table 1). The use of a RNA virus that does not enter the nucleus as Sendai virus, allows faster viral clearance with a complete elimination of all viral RNA from the tenth passage (Supplementary Table 2) and were thus cleared of the four transgenes (OSKM) when analyzed by CGH arrays.

**Figure 1:**
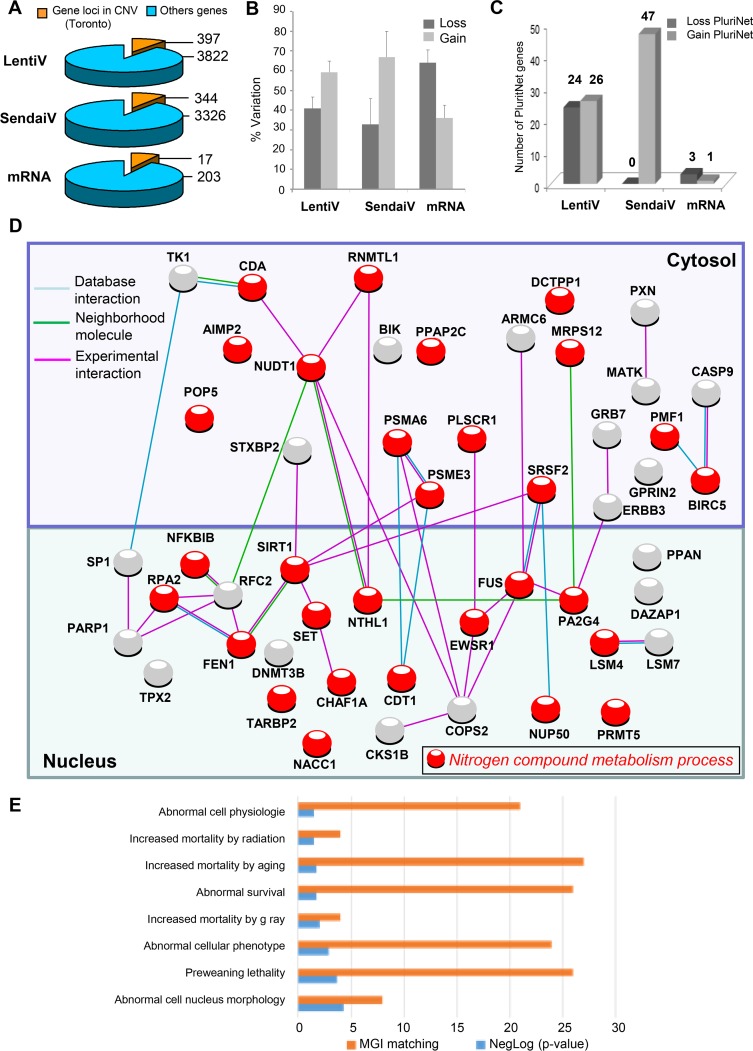
PluriNet and Mammalian phenotype analysis performed on genomic alterations of integrative hiPSCs (**A**) Pie charts of gene locus alterations observed by aCGH technology on genomic DNA of hiPSCs cells: the yellow part represents gene loci that are known as to be polymorphism CNV in Toronto database and the blue part represents gene loci which passed the polymorphism filtration. (**B**) variation type of CNV (gain/loss). (**C**) Histogram of loss and gain CNVs observed by different hiPSCs methods affecting gene locus present in PluriNet database. (**D**) Pluripotent stem-cell-specific protein-protein interaction network detected by PluriNet database in genomic aCGH alterations from integrative hiPSCs: Nucleus and cytosolic cellular compartments are separated on the network, gene locus alterations are represented by nodes and protein-protein interactions by links obtained with STRING10 application (blue link: protein interaction database source; pink link: experimental interaction, green link: neighborhood molecule), red nodes represent genes belonging to the most enriched biological process on gene ontology database: nitrogen compound metabolism process. (**E**) Analysis of PluriNet genomic aCGH alterations from integrative hiPSCs in the context of MGI-mouse phenotype which predict Mammalian phenotype (blue bars of the histogram represent enrichment negative base 10 logarithm of *p*-value with Benjamini, Hochberg correction for multi-testing, orange bars represent number of MGI mouse phenotype which matched with alterations).

As expected [1, 2] we found less CNVs when a mRNA transfection method was used with the detection of a total of 83 CNVs (Supplementary Figure 2A) for the 3 cell lines tested (9 CNS per iPSCs, with 20, 36 and 27 CNVs) containing a total of 203 different altered gene loci (67 genes per iPSCs) (Figure 1A). By using Sendai virus a total of 157 different CNVs were identified for the 3 iPS lines tested (17 CNVs per iPSCs, with 58, 85 and 14 CNVs) (Supplementary Figure 2A) containing a total of 3326 different altered gene loci (Figure 1A) corresponding to 1108 genes per iPSCs. The use of the integrative method has generated 8.8 CNVs per iPSCs (range 10–97) affecting for the 6 iPSCs tested a total of 3822 different gene loci (Figure 1A) corresponding to 1108 genes per iPSCs. We were not able to observe significant differences between the percentages of DNA losses or DNA gains between both viral methods (Figure 1B), affecting mainly small chromosomes such as chromosomes 17 and 19 as well as genes involved in the G2M cell cycle transition (Supplementary Figure 3).

**Figure 2:**
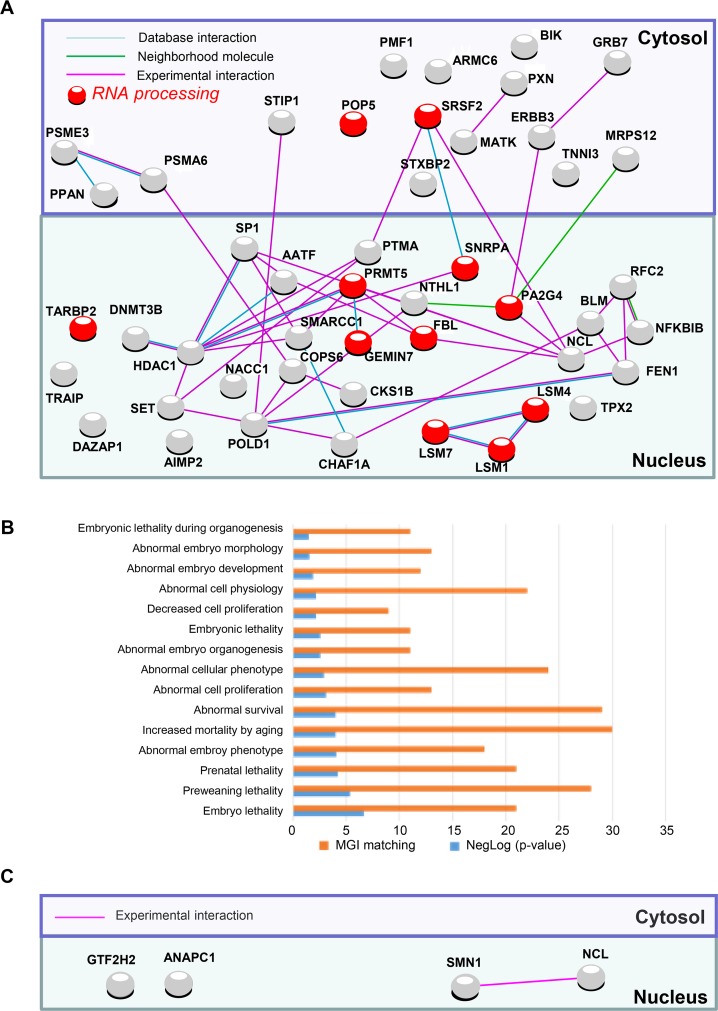
PluriNet and Mammalian phenotype analysis performed on genomic alterations of non-integrative hiPSCs (**A**) Pluripotent stem-cell-specific protein-protein interaction network detected by PluriNet database in genomic aCGH alterations from hiPSCs generated with Sendai viruses: Nucleus and cytosolic cellular compartments are separated on the network, gene locus alterations are represented by nodes and protein-protein interactions by links obtained with STRING10 application (blue link: protein interaction database source; pink link: experimental interaction, green link: neighborhood molecule), red nodes represent genes belonging to the most enriched biological process on gene ontology database: RNA processing. (**B**) Analysis of PluriNet genomic aCGH alterations from Sendai derived-hiPSCs in the context of MGI-mouse phenotype which predict Mammalian phenotype (blue bars of the histogram represent enrichment negative base 10 logarithm of *p*-value with Benjamini, Hochberg correction for multi-testing, orange bars represents number of MGI mouse phenotype which matched with alterations). (**C**) Pluripotent stem-cell-specific protein-protein interaction network detected by PluriNet database in genomic aCGH alterations from mRNA derived-hiPSCs: Nucleus and cytosolic cellular compartments are separated on the network, gene locus alterations are represented by nodes and protein-protein interactions by links obtained with STRING10 application (pink link: experimental interaction).

In order to verify whether these CNVs can affect the pluripotency we first matched them with the Pluripotency associated Network (PluriNet) [5] and we thus identified 50 and 47 gene loci alterations (DNA losses or gains) linked to PluriNet when lentivirus and sendai viruses are used respectively (Figure 1B). The 47 alterations linked to Plurinet were exclusively gained in Sendai viruses iPS lines affecting mainly the small chromosomes such as 17 and 19 (Supplementary Figure 4). Surprisingly, 33 of these alterations were found common to both viral methods (Supplementary Figure 5). Out of the 33 common DNA alterations 26 were gained and 7 were lost. Concerning the integrative method the alterations implied proteins located both in the cytoplasm (24 alterations) and in the nucleus (26 alterations) (Figure 1C and Supplementary Table 3) and were linked to abnormal cell morphology in MGI database (Figure 1E). Integrative methods were found to alter several important pathways including mostly the nitrogen compound metabolic process (Figure 1D), DNA replication and Base excision repair functions (Supplementary Table 3) in contrast to Sendai virus method affecting mostly nuclear proteins (Figure 2A and Supplementary Table 2) implying in the machinery of mRNA including the RNA processing and splicing, the spliceosome and the RNA degradation processes (Figure 2A and Supplementary Table 4). and in embryo development in MGI database (Figure 2B). Concerning the hiPSCs produced with mRNA only 4 genes were altered (3 losses and 1 gain) encoding 4 different nuclear proteins (Figure 2C).

**Figure 3:**
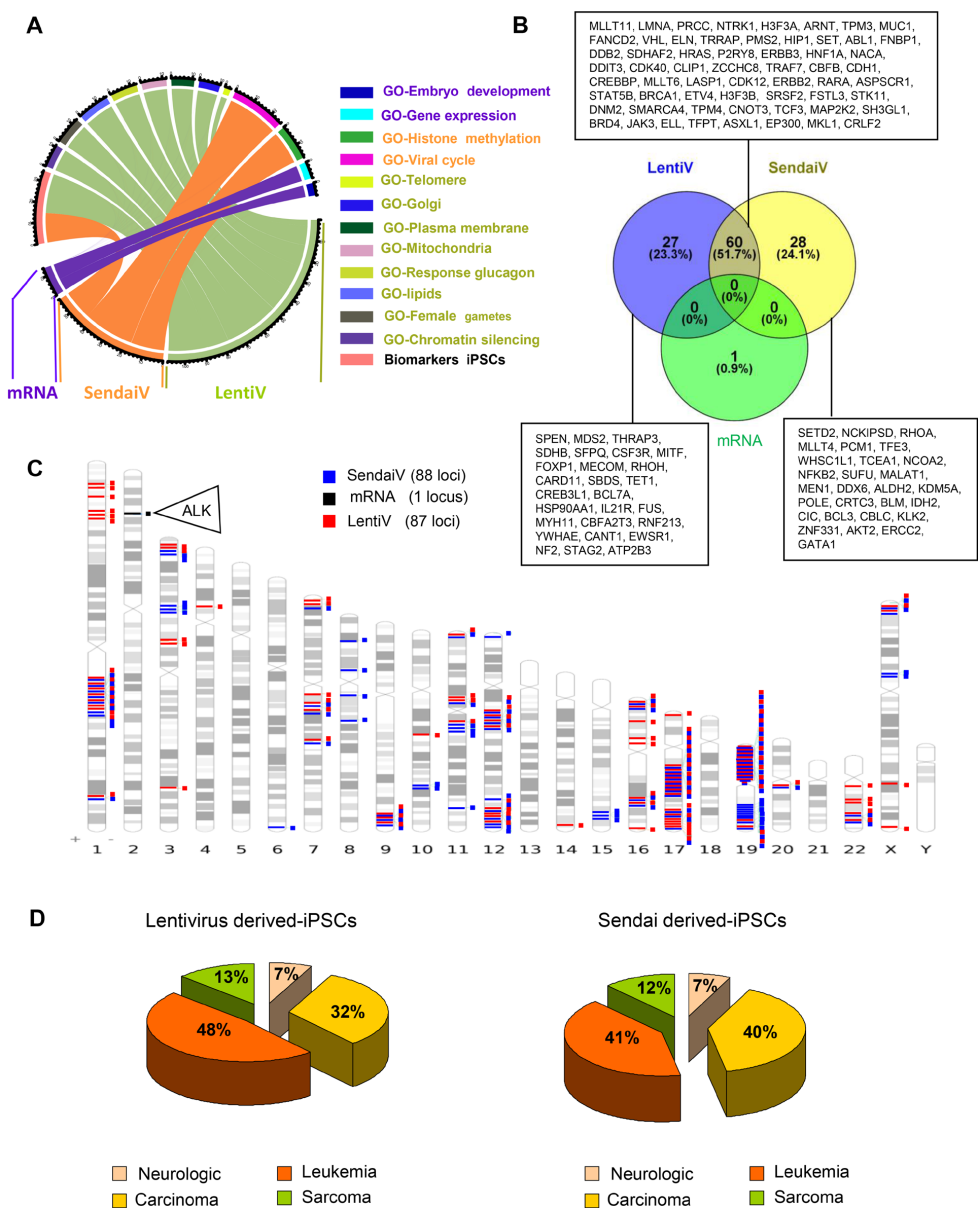
Cancer related gene locus found to be altered by aCGH in hiPSCs cells (**A**) Circos plot of functional enrichment performed on gene ontology-biological process and Biomart-biomarkers databases with gene locus aCGH alterations obtained on hiPSCs cells with the 3 methods: lentivirus, Sendai virus and mRNA. (**B**) Venn diagram of cancer related genes found in COSMIC database with aCGH genomic alterations from hiPSCs cells performed with 3 different methods: lentivirus, Sendai virus and mRNA. (**C**) Karyotype ideogram of cancer related genes found in COSMIC database with aCGH genomic alterations from hiPSCs cells performed with 3 different methods: lentivirus (red), Sendai virus (blue) and mRNA (black). (**D**) Pie charts of oncogenic categories for aCGH genomic alterations from hiPSCs cells obtained by Lentivirus and Sendai virus methods.

We then merged and filtered each set of gene loci comprised in detected CNVs with the software GO-Elite Standalone version 1.2.5 [7] by using Biomarkers and Gene Ontology Biological Process databases (http://www.genmapp.org/go_elite/help_main.htm). As seen on the CircosPlot representation, 20 and 15 hiPSCs biomarkers were significantly affected (*p* < 0.001) only in virally transduced hiPSCs (Figure 3A and Supplementary Table 5) and 88, 90 and 14 markers were found significantly (*p* < 0.05) deregulated after filtration with Gene Ontology Biological Process database respectively for LentiV, SendaiV and mRNA-derived hiPSCs (Figure 3A and Supplementary Table 4).

In order to verify whether each set of gene locus alterations in CNVs present a tumorigenic risk, each set was then filtered with COSMIC program. We found only one cancer-associated damage in hiPSCs produced by mRNA (ALK) whereas more than 80 different cancer-associated damages were revealed after viral transduction (Figure 3B–3C and Supplementary Table 6), mostly located on chromosomes 1, 12, 17 and 19 (Figure 3C). After filtering them with gene loci known to be implicated in human malignant tissues [6] we found that the majority of these alterations in CNVs were known to be deregulate in several types of leukemia (41 to 48%) and carcinoma (32 to 40%) (Figure 3D and Supplementary Figure 6).

### Analysis of genomic integrity in teratoma derived from of hiPSCs produced by three different reprogramming strategies

In order to evaluate the CNV rates after differentiation we generated teratomas *in vivo* by injecting intramuscularly 2 × 10^6^ iPSCs into NOD-SCID/gnull mice. For this purpose we chose 3 iPSCs for which CNVs analysis has been also carried out on the iPSCs used to perform the teratoma as well as on original cells using the same batch of chip. By using these criteria the CNVs analysis could be rigorously performed by excluding the CNVs observed in original cells and iPSCs in order to determine only the CNV that could appear in the teratomas after differentiation. In addition, we selected 3 iPSCs with a complete viral clearance for which the 4 transgenes used for reprogramming could not be detected by qRT-PCR.

**Figure 4:**
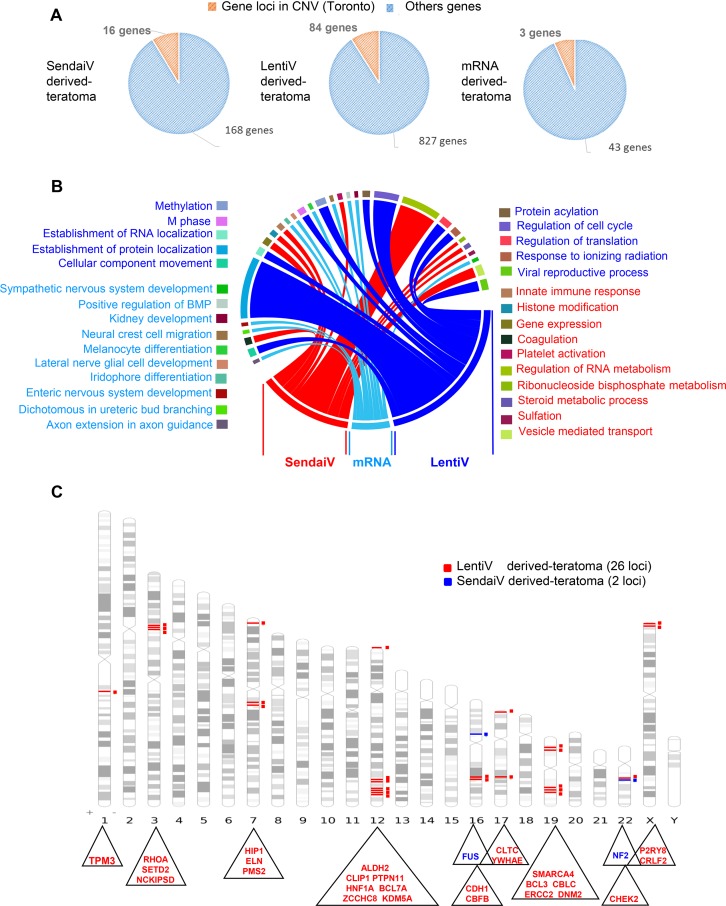
Cancer related gene locus found to be altered by aCGH in teratomas (**A**) Pie charts of gene locus alterations observed by aCGH technology on genomic DNA of teratoma: the orange part represents gene loci that are known as to be polymorphism CNV in Toronto database and the blue part represents gene loci which passed the polymorphism filtration. (**B**) Circos plot of functional enrichment performed on gene ontology-biological process database with gene locus aCGH alterations obtained on teratoma with the 3 methods: lentivirus, Sendai virus and mRNA. (**C**) Karyotype ideogram of cancer related genes found in COSMIC database with aCGH genomic alterations from teratoma performed with lentivirus (red), Sendai virus (blue) methods.

After 60 days of differentiation, teratoma were removed and pathologically analyzed. All teratomas showed a differentiation into ectodermal, endodermal and mesodermal tissues (data not shown). In contrast to teratoma derived from iPSCs produced by Sendai virus and mRNA, large areas of malignant tumors were present in the teratoma produced by lentiviral integrative method, displaying typical features of invasive carcinomas. As expected, the highest rates of CNV were found for lentivirus-derived teratoma showing malignancy (total of 88 CNVs; 7 gains and 81 losses) compared to Sendai-derived teratoma (total of 17 CNVs; 12 gains and 5 losses) and mRNA-derived teratoma (total of 12 CNVs corresponding all to a gain status) containing a total of 827, 168 and 43 different altered gene loci respectively (Figure 4A). The CNVs were found to be linked with at least ten different cell functions (Figure 4B). Each set of gene loci comprised in detected CNVs was then merged and filtered with COSMIC and cBioPortal (http://www.cbioportal.org) programs. After differentiation there was a trend towards fewer gene locus variations linked to cancer. Indeed, we identified up to 26 and 2 (FUS and NF2) loci when reprogrammed cells were produced with Lentiviral and Sendai methods respectively and no cancer associated-gene locus variations were found in teratoma generated with mRNA-derived hiPSCs (Figure 4C, Supplementary Table 6). We then merged these loci with cBioPortal program allowing access to cancer genomics data sets from human tumor samples from different cancer studies. Thus, all 26 loci found in lentivirus-derived teratoma were found to be altered in a large number of cancers including lung carcinoma or stomach adenocarcinomas and to be significantly (*p* = 0.02) associated with poor survival [8, 9] (Supplementary Figure 7).

### Histological analysis of teratoma produced by integrative and non-integrative vectors

**Figure 5:**
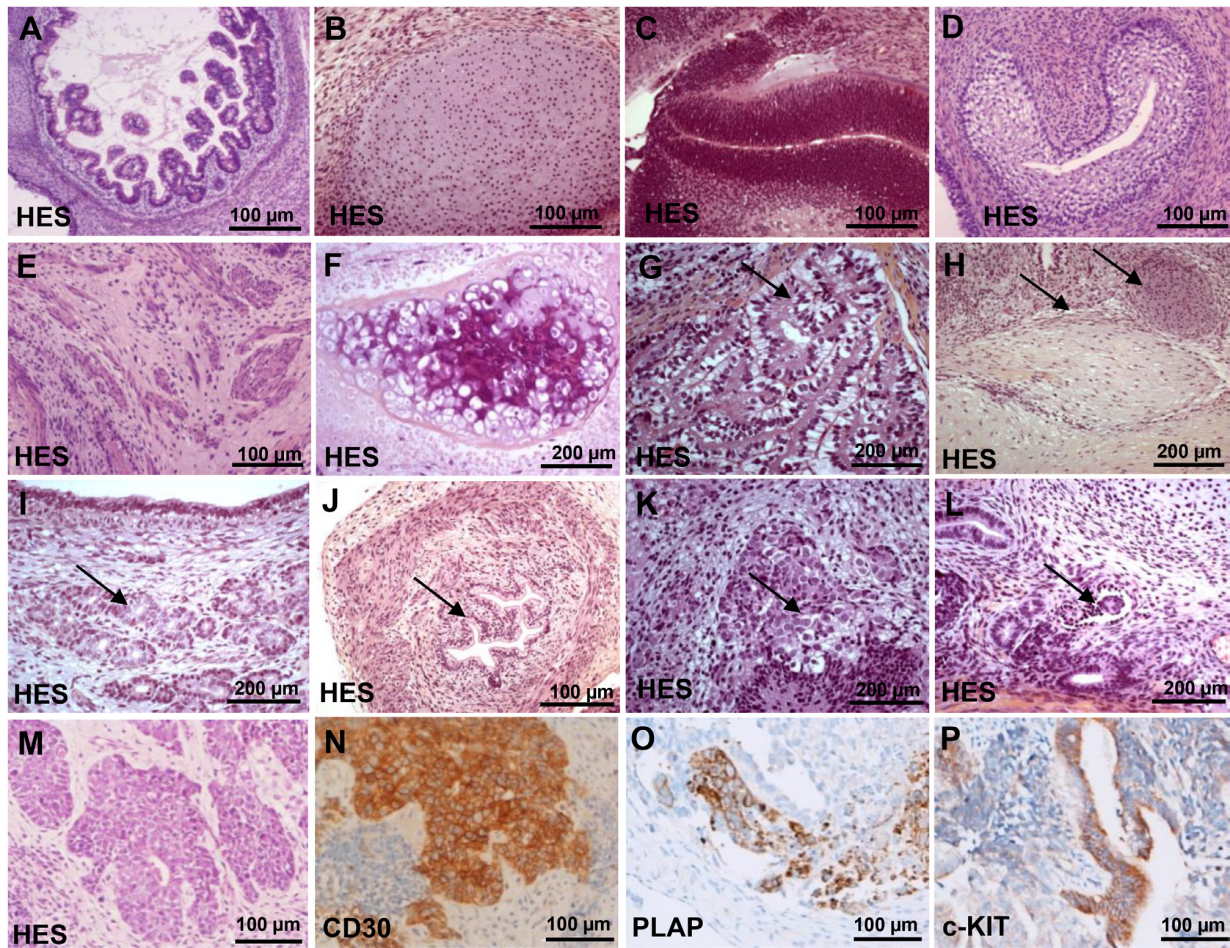
Histological analysis of teratomas by haematoxylin and eosin staining (HES) (**A**) intestinal epitheliums. (**B**) cartilaginous areas. (**C**) muscle. (**D**) bone structures. (**E**) glial tissues and neural crest. (**F**) malpighian epitheliums. (**G**) plexus choroid. (**H**) peripheral nerves and fatty tissue. (**I**) bronchial glands. (**J**) urinary epithelium with a muscular wall. (**K**) neuroendocrine tissue. (**L**) glomerular like structure. (**M**) Teratoma histological analysis showing malignant tumor by haematoxylin and eosin staining (HES). (**N**) Immunohistochemistry for CD30. (**O**) Immunohistochemistry for PLAP. (**P**) Immunohistochemistry for c-kit.

To evaluate the pluripotency of each hiPSCs and to determine whether they could potentially give rise to malignant tissues *in vivo* we generated 50 teratomas from hiPSC generated with either lentivirus (*n* = 21), Sendai virus (*n* = 19) and mRNA (*n* = 2) and compared them to teratomas derived from hESCs (*n* = 8). All hiPSCs produced teratomas after 52 to 102 days. Histological analysis (Figure 5A–5L) of all teratomas showed a differentiation into ectodermal, endodermal and mesodermal tissues, mainly represented by malpighian epitheliums (71 to 100%), glial tissues (79 to 100%), intestinal epitheliums (90 to 100%), bone structures (62 to 100%) and large cartilaginous areas (75 to 100%) (Supplementary Table 7). In 8 out of 50 teratomas performed, malignant tissues have exclusively emerged in teratomas generated by hiPSCs produced by integrative methods and recovered at low passage (mean of 12 ± 5). Five iPSCs were generated with OSLN cocktail and three with OSKC cocktail (Supplementary Table 10). All teratomas have shown typical features of invasive carcinomas including an irregular multi-layered epithelium consisting of tumor cells with increased nuclear-cytoplasmic ratio, pleomorphic and hyperchromatic nuclei (Figure 5M). The 5 teratomas generated with OSLN-derived iPSCs were immune-reactive for CD30 (Figure 5N) placental alkaline phosphatase (PLAP) (Figure 5O) and for c-kit (Figure 5P)and the 3 teratomas generated with OSKC have revealed the presence of large areas of carcinomas by HES staining positive for c-Myc (Supplementary Figure 8). This latter result should be due to residual c-Myc transgene expression and/or the reactivation of the c-Myc transgene during teratoma development.

### Characterization of CNV levels in hiPSCs and teratoma generated with integrative vectors

**Figure 6:**
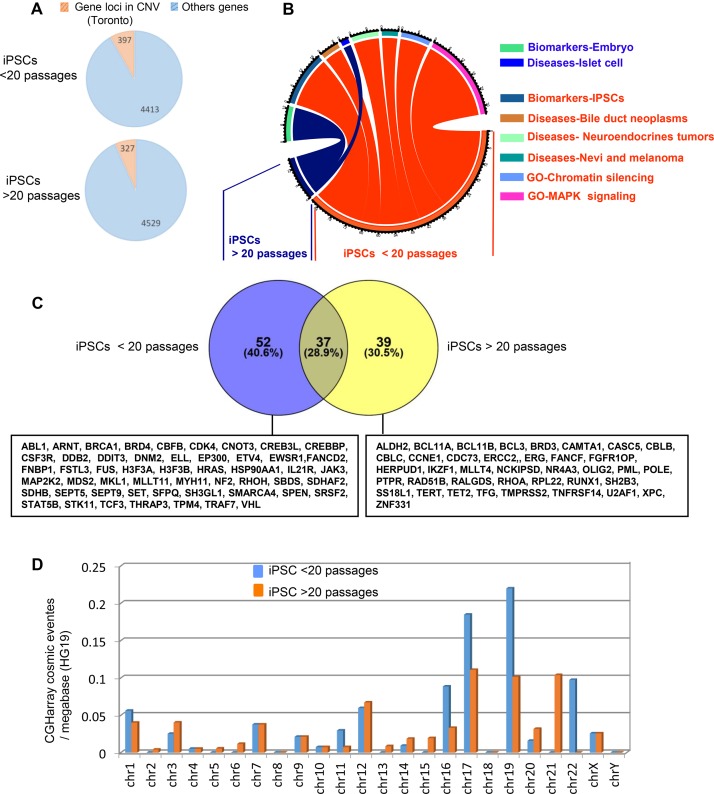
Passage number rate integration analysis of cancer related gene locus found to be altered by aCGH in hiPSCs (**A**) Pie charts of gene locus alterations observed by aCGH technology on genomic DNA of hiPSCs taking into account the number of passages (less or more than 20 passages: the orange part represents gene loci that are known as to be polymorphism CNV in Toronto database and the blue part represents gene loci which passed the polymorphism filtration. (**B**) Circos plot of functional enrichment performed on gene ontology-biological process, CTD-diseases and Biomart-biomarkers databases with gene locus aCGH alterations obtained on hiPSCs taking into account the number of passage (less or more than 20 passages). (**C**) Venn diagram of cancer related genes found in COSMIC database with aCGH genomic alterations from hiPSCs cells comparing low and high number of passages. (**D**) histogram of COSMIC events by chromosome in aCGH for hiPSCs cells (low and high number of passages), events are normalized by megabase on version HG19 of human genome.

To explain the occurrence of aggressive tumors within the lentivirus derived-teratomas we have analyzed and compared the CNV rates in hiPSCs at different passages in the corresponding teratomas. The mean number of CNVs was not significantly higher in the early passage of hiPSCs (14 ± 4, *n* = 6) compared to late passage (36 ± 7, *n* = 6) with respectively of a mean 52 and 53 CNS (range 10–97) (Supplementary Figure 2B) containing a total of 4413 and 4529 different altered gene loci (Figure 6A). Functional enrichment analysis of those genes on Gene Ontology Biological Process, Biomarkers and Diseases databases were shown to specifically affect hiPSCs signatures, embryo development and to neoplastic diseases such as Bile duct neoplasms, neuroendocrine tumors, melanoma, and pancreatic islet cell tumors (Figure 6B and Supplementary Table 8). Functional enrichment on the COSMIC database revealed a large number of altered genes linked to tumor for both groups with 89 and 76 altered gene loci in early and late passage hiPSCs respectively (Figure 6C) affecting mostly short chromosomes (chromosomes 16, 17, 19 21 and 22) (Figure 6D and Supplementary Table 9).

**Figure 7:**
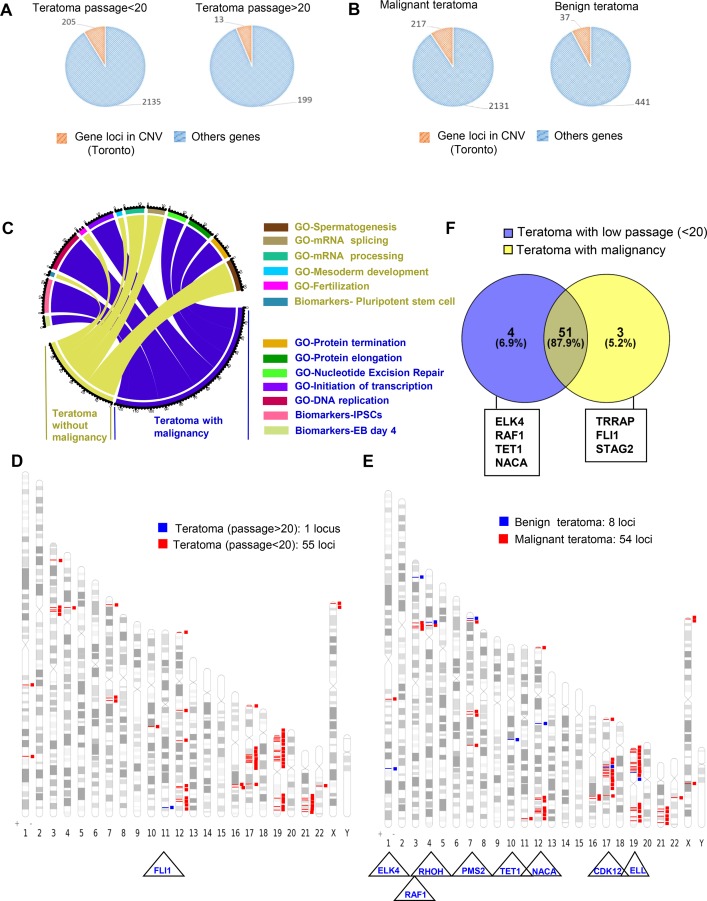
Passage number integration analysis of cancer related gene loci found to be altered by aCGH in teratomas (**A**) Pie charts of gene locus alterations observed by aCGH technology on genomic DNA of teratoma taking account of hiPSCs passage number (<20 or >20 passages): the orange part represents gene loci that are known as to be polymorphism CNV in Toronto database and the blue part represents gene loci which passed the polymorphism filtration. (**B**) Pie charts of gene locus alterations observed by aCGH technology on genomic DNA of teratoma taking account of teratoma malignancy status: the orange part represents gene loci that are known as to be polymorphism CNV in Toronto database and the blue part represents gene loci which passed the polymorphism filtration. (**C**) Circos plot of functional enrichment performed on gene ontology-biological process and Biomart-biomarkers databases with gene locus aCGH alterations obtained on teratoma taking account of malignancy status. (**D**) Venn diagram comparing cancer related gene locus aCGH altered in teratoma with low number of passages (<20) and in teratoma with malignancy. (**E**) Karyotype ideogram of cancer related genes found in COSMIC database with aCGH genomic alterations from teratoma taking into account the number of passages (blue: more than 20 passages, red: less than 20 passages). (**F**) Karyotype ideogram of cancer related genes found in COSMIC database with aCGH genomic alterations from teratoma taking into account the malignancy status of teratoma (blue: benign, red: malignant).

In contrast to hiPSCs, the mean number of gene loci alterations included in the CNVs was tenfold lower in teratoma generated with late passage hiPSCs compared to teratoma generated with hiPSCs injected at early passage (199 versus 2135 gene loci included in the CNVs, corresponding to respectively 3.6 and 3.4 CNVs per teratoma (Figure 7A). Using the same functional enrichment database as previously for iPSCs, these events affected mostly RNA metabolism (including RNA splicing and catabolism) and nucleotide-excision repair system for the teratomas generated with iPSCs at lower passage and spermatogenesis, RNA splicing and processing for the teratomas generated with iPSCs at later passage (Supplementary Table 8).

In addition, we found that the mean number of gene loci inside the CNV rates were fivefold lower in benign teratoma as compared to teratoma with malignant areas (441 versus 2131 gene loci corresponding to respectively to a mean of 29 (range 8-88 with 2.3 CNVs par teratoma) and 11 (range 5–18 with 5 CNVs per teratoma) CNVs (Figure 7B, Supplementary Figure 2D) affecting respectively important cell functions including the machinery of mRNA processing and splicing as well as the machinery of proteins, the DNA replication and the Nucleotide Excision Repair system (Figure 7C, Supplementary Table 6).

In order to find a genomic signature associated with cancer, all sets of gene loci inside the CNVs were annotated with COSMIC database showing that the number of cancer-associated genes were much higher in teratoma generated with early passage hiPSCs (55 versus 1 loccus) (Figure 7D and Supplementary Table 7) and in malignant-associated teratoma (54 versus 8 locus) (Figure 7E and Supplementary Table 7) compared to those generated with higher passage and to those that have generated benign teratoma (Figure 7E). From the CNV sets found in hiPSCs-derived teratomas (55 genes) and in malignant derived teratoma (54 genes) we performed a Venn diagram showing that 87.9% of these alterations were common to both groups of teratoma (Figure 7F).

## DISCUSSION

The genome of hiPSCs are known to be intrinsically unstable with the potential ability to generate potentially hazardous genomic aberrations [1, 10, 11], probably resulting from various mechanisms including a replicative stress [1], the reactivation of the telomerase [12], the metabolism modification from the oxidative to the glycolytic state [13] or from the perturbation of cheekpoints or repair of DNA double strand breaks leading to non-allelic homologous recombination (NAHR)-based rearrangements and/or non-homologous end-joining (NHEJ)-based rearrangements [14]. All these processes are shared with the mechanisms activated during oncogenesis and therefore it is possible that some of these events can also be associated with the generation of an oncogenic event during the reprogramming. Some alterations could affect tumor-suppressor genes or oncogenes, dramatically affecting the quality of the final product. This has been reported in several studies with hiPSCs generated with viral integrative methods, demonstrating the emergence of oncogenes during either the late phase or the early phase of hiPSCs establishment including genes such as RHOC, NRAS, AKT3, MDM2, CTAGE4 [10] or MYC, RAS, p53, ERBB3 [1]. These observations contrast with a recent study where the genome-wide mutation rates were assessed in hiPSCs generated by three distinct methods including, retrovirus, Sendai virus and mRNA for which no mutations known to be associated with increased cancer risk were identified [15].

In this work we have generated a combined analysis of 42 hiPSC generated by three methods and analyzed genomic integrity of these cells in a pluripotent stage as well as after their long-term differentiation using a teratoma model. We show here that the reprogramming process is associated with higher genomic alterations when hiPSCs are generated with viruses compared to hiPSCs generated with mRNA. Interestingly only gain events were observed in Sendai reprogrammed iPSCs that were mainly observed on small chromosomes like the 17 and the 19. These events were also observed on the long arm of these two small chromosomes, so they didn’t concern the frequent 17p rearrangement comprising Tp53 which is usually observed during carcinogenesis. Gains on the long arm of chromosome 19 comprised events such as in region q13.41-q13.42 which implicated clusters of miRNA Genomic precursors and effectively miRNA could implicate large transcriptional program regulations as observed during development. Gains on the long arm of chromosome 17 comprised events such as in region q21.2-21.3which comprised the gene locus of BRCA1 known to be implicated in DNA repair and in the development of familial breast carcinogenesis. This same genomic region affected also the ETV4 locus which is a DNA-binding transcription factor and potentially interacts with viruses: Polyomavirus Enhancer Activator 3 Homolog or Adenovirus E1A Enhancer-Binding Protein.

In addition only hiPSCs generated with mRNA appeared reliable since they were shown to share at 98.6% the same protein-protein network (“PluriNet”) with bona fide pluripotent cells as previously described [5].

We also conclude that all reprogramming cells have potentially cancer associated alterations independently to the methods used. Nevertheless, these alterations were found mainly in viral transduced-hiPSCs and were found to be mostly linked to those found in a wide type of carcinoma and leukemia. Only one alteration in the anaplastic lymphoma kinase (ALK) locus was found in hiPSCs generated with mRNA that was shown to predispose to neuroblastoma [16] colorectal adenocarcinoma [17] and non-small-cell lung carcinoma [18].

For viral transduction methods cancer-associated genomic alterations were mostly found on 18 different chromosomes except chromosomes 5, 13 18 and 21. We identified 87 and 88 different potentially tumorigenic altered loci in viral derived-hiPSCs and 60 CNV (51.7%) were found common to both viral strategies. That was the case for BRCA1, ERBB2 and ERBB3 loci that were found to be deregulated in triple-negative breast cancers [19] or von Hippel-Lindau (VHL) tumour suppressor gene and PRCC that was linked to clear cell renal cell carcinoma and papillary renal cell carcinoma respectively [20, 21]. Others loci were altered such as BRD4, EP300, H3F3A and H3F3B involved in chromatin remodeling recreating, when mutated, a hallmark of chondroblastoma and giant cell tumor of bone [22] in addition to JAK3, ASXL1 and ELL that confers, once altered, features of megakaryoblastic leukemia [23], myelodysplastic syndromes [24] and acute promyelocytic leukemia [25] respectively. The proto-oncogene HRAS was also found altered in both cell lines that have the potential to cause a Costello syndrome or bladder, thyroid or salivary duct carcinoma [26] as well as HIP1 and SDHAF2 an early-stage prognostic biomarker of lung adenocarcinoma [27] and a hallmark of pheochromocytomas respectively [28].

We then identified 28 different potentially tumorigenic loci in hiPSCs generated with Sendai vectors such as MALAT1, an oncogenic long non-coding RNA (lncRNA) originally identified in non-small cell lung tumors conferring a high risk of metastasis [29]. MALAT1 has been implicated in alternative splicing regulation, in transcriptional control of genes involved in cell cycle, cell motility and EMT [30] and could act as a transcription activator by mediating assembly of Polycomb repressive complexes [31]. PCM1 was also altered which is associated with papillary thyroid carcinomas and a variety of hematological malignancies, including atypical chronic myeloid leukemia and T-cell lymphoma [32, 33]. Other loci were found altered such as Aldehyde dehydrogenase 2 (ALDH2) and isocitrate dehydrogenase 2 (IDH2), that was found to predispose to cervical carcinoma [34] and glioma [35] respectively as well as BCL3, AKT2 and ERCC2 for which polymorphisms were shown to be associated with lung carninoma [36–38].

We also identified 27 different potentially tumorigenic loci in hiPSCs generated with Lentiviral vectors. That was the case for Neurofibromatosis Type II (NF2) and STAG2 that was shown to predispose for MISME (“Multiple Inherited Schwannomas, Meningiomas, and Ependymomas”) Syndrome [39] and Ewing sarcoma [40] respectively. Others loci are associated with hematological disorders such as CEBPA, FOXP1, MECOM and RHOH which promotes Acute myeloid leukemia [41], diffuse large B-cell lymphoma [42], myeloproliferative neoplasm [43] and B-cell chronic lymphocytic leukemia [44] respectively.

All hiPSCs, independently of the method used, have given rise to teratoma containing tissues belonging to all three germ layers despite the alteration of important cellular functions identified with four different databases. The global altered gene loci were reduced by 78 to 95% in teratoma compared to the hiPSCs counterpart in addition to those associated with a tumorigenic risk. No cancer-associated alterations could be detected in teratoma derived from mRNA-derived hiPSCs and only two genes (FUS, NF2) were found altered in Sendai-derived teratoma that were associated with human sarcoma. FUS/TLS (fused in sarcoma or translocated in liposarcoma) was identified as a translocated gene in human liposarcoma and leukemia [45] and NF2 in human neurofibrosarcoma and schwannoma [46].

In contrast we identified more than 50 different loci of cancer-associated alterations mainly in teratoma generated with early passage (<20) of hiPSCs produce by integrative vectors which results in the emergence of malignant tumors within the teratoma such as an invasive carcinomas positive for c-Myc or for PLAP and c-kit which are frequently associated with gastrointestinal stromal tumors [47] and melanoma [48].

In summary our study demonstrates that the mRNA reprogramming strategy yields hiPSCs that appears to have the more similar protein-protein network to that previously described in “PluriNet” in contrast to hiPSCs generated with integrative or non-integrative viral strategies showing 50 and 47 different alterations which showed no disturbance of their pluripotency property. Nevertheless, all three reprogramming strategies can lead to the occurrence of CNV that are associated with a tumorigenic risk, but there were subtle differences among the methods. We found that the non-integrating mRNA reprogramming technique resulted in extremely rare occurrence of cancer-associated CNV gene loci alteration either in an undifferentiated state or after long-term differentiation, compared to others methods conducive to the occurrence of numerous hazardous CNV that are also found deregulated in different types of carcinoma and leukemia. This highlights the need for careful studies of cancer-associated genomic alterations to select hiPSC lines with no potential tumorigenic risks, even though the non-integrative and non-viral technology appears safer for applying stem cell-based therapies for human disease.

## MATERIALS AND METHODS

### Cell culture

Mouse embryonic fibroblasts (MEF) obtained from E13.5 mouse embryos (Harlan, CD1-ICR strain) were expanded in MEF medium containing DMEM High Glucose with glutamax supplemented with 15% fetal bovine serum, 1% non-essential amino acids, 1% penicillin-streptomycin, 0.1 mM 2-mercaptoethanol (all of them from Lifetechnologies). For inactivation, MEF were incubated overnight in MEF medium supplemented with 1 μg/ml Mtiomycin-C (Sigma-Aldrich, catalogue number M4287). Human foreskin fibroblasts (Millipore, catalogue number SCC058) were cultured in FibroGRO™-LS Complete Media Kit (Millipore, catalogue number SCMF001). For lentiviral and sendaï virus reprogramming, iPSCs were derived and maintained in hESC medium containing DMEM/F12 supplemented with 20% Knock Out Serum Replacement, 0.2 mM L-glutamine, 1% non-essential amino acid, 1% penicillin-streptomycin, 0.1 mM 2-mercaptoethanol (all of them from Lifetechnologies) and 12.5 ng/ml human recombinant basic Fibroblast Growth Factor (Miltenyi Biotec, catalogue number 130-093-843). For mRNA reprogramming, iPSCs were derived in Pluriton medium (Miltenyi Biotec, catalogue number 130-096-820) and then adapted to hESC medium for expansion. SSEA4 and Tra-1-60 immuno staining and Karyotype were performed ad previously described [49]. All iPSCs characteristics are listed on Supplementary Table 10.

### Lentiviral reprogramming

Human foreskin fibroblasts (Millipore, catalogue number SCC058) were reprogrammed using the Human STEMCCA Constitutive Polycistronic Lentivirus Reprogramming Kit (Millipore, catalogue number SCR544) according to the manufacturer’s instructions. Four weeks after transduction, colonies were manually picked and expanded onto Mitomycin-C (Sigma) inactivated mouse embryonic fibroblasts in human embryonic stem cell medium (hESC medium).

### mRNA reprogramming

Human foreskin fibroblasts (Millipore, catalogue number SCC058) were reprogrammed using the Stemgent mRNA Reprogramming Factors Set (Miltenyi Biotec, catalogue number 130-096-528) according to the manufacturer’s instructions. Two weeks after the beginning of transfection, colonies were manually picked and expanded onto Mitomycin-C inactivated mouse embryonic fibroblasts in hESC medium.

### Sendaï virus reprogramming

Human forskin fibroblasts were reprogrammed using the Cytotune-iPS Sendaï Reprogramming Kit (Lifetechnologies, catalogue number A1378001) as previously described [50].

### Reverse transcription-PCR

Two different methods were used to detecte the presence of the four transgenes after rthe reprogramming process. The transgenes used to derive lentiviral-derived iPSCs were quantified by Real-Time Reverse Transcription-PCR using the ABI PRISM 7900 Sequence Detection System as previously described [49]. The transgenes used to derive sendai-derived iPSCs were quantified in accordance with the guidelines provided by the Cytotune-iPS Sendaï Reprogramming Kit by using RNA Extraction: TRIzol™ Reagent (Invitrogen, catalogue number 15596-026), RT reaction: SuperScript™ VILO™ cDNA Synthesis Kit (Invitrogen, catalogue number 11754-050) and the PCR: AccuPrime™ SuperMix I (Invitrogen, catalogue number 12342-010).

### Teratoma formation and immunohistochemistry

The teratoma assay was performed with ESCs and iPSCs by i.m. injection of 1 to 3 × 10^6^ cells into 6-week-old NOD/SCID mice (Charles River Laboratories, Lyon, France). After 5 to 10 weeks, teratomas were dissected and fixed in 4% paraformaldehyde and samples were embedded in paraffin and stained with H&E in association with IHC, to assess the presence of ectodermic, endodermic, and mesodermic tissues. The IHC was performed as requested with a Benchmark XT apparatus (Ventana Medical System, Illkirch, France) with prediluted primary antibodies raised against placental alkaline phosphatase (PLAP), c-Myc and ckit (Dako, France).

### Oligonucleotide based-array CGH 135K analysis

Genomic imbalances were analyzed according to the manufacturers protocols with the human CGH 12 × 135K Whole-Genome Tiling v3.0 Array, (Roche NimbleGen, Meylan, France). Briefly, genomic DNA (0.5 μg) was fluorescently labeled with the Roche NimbleGen Dual-Color DNA labeling kit (Roche NimbleGen). We used a Dye-swap procedure and human male genomic DNA as a reference (provided by Promega, Charbonnière, France). The iPSC line DNA and the sex-matched reference DNA were denatured before hybridization for 48 h at 42° C (NimbleGen Hybridisation kit) using the Hybridization System 4 (Roche NimbleGen). The slides were then washed (NimbleGen Wash Buffer Kit), and scanned on a Roche NimbleGen MS200 Microarray Scanner. All the captured images were processed with NimbleScan software and data analysis was performed with DEVA software v1.0.2 (Roche Nimblegen). Statistical analysis was performed using the Nexus Copy Number Standard edition software algorithm (Proteigene, Saint-Marcel, France), with build 18 of the human genome and subsequently liftover in Hg19 genomic coordinates (http://genome.ucsc.edu/). Quantitative ratios obtained by CGHarray technology were integrated in multi-experimental matrix by SQL query. Heatmaps sorted by chromosome were realized by using MEV software version 4.9.0. Multi-experimental matrix were also transformed in files with *.gct extension compatible with a visualization in Integrative Genomics Viewer (IGV) software version 2.3.32 [51]. Copy number variation (CNV) polymorphisms were filtered from the matrix with web application SCANDB (http://www.scandb.org) on the population CEPH (North and Western Europe - include CNVs that predict expression with *p*-value less than *p*-value *p* < 0.0001 for population CEU [52]. Functional enrichment analyses were performed on filtered matrix with the software GO-Elite Standalone version 1.2.5 7 by using database Biomarkers and Gene Ontology Biological Process. In order to found CNVs loci which touched genes implicated in cancer, the CGHarray processed matrix were merged and filtered with COSMIC database: Catalogue of somatic mutations in Cancer (http://cancer.sanger.ac.uk/census) [6]. As CGHarray experiments were also performed to study human pluripotente reprogramming cells CGHarray results were matched with genes belonging to PLURINET network of pluripotency [5]; Muller *et al* list of 299 genes, was found in MSigDb version 5.1 database at the address: (http://software.broadinstitute.org/gsea/msigdb/cards/MUELLER_PLURINET). Interaction networks on molecules linked to the pluripotency were performed with the application STRING version 10.0 web by retaining intermolecular connections listed in protein interaction and experiment databases [53]. Mouse phenotypes were predicted on genes found altered in CGHarray and linked to pluripotency with the application MouseMine [54]. The altered CNVs from PluriNet were thus analyzed in the context of phenotypes, which have been reported to result from specific genetic manipulations (e.g. gene knock-out) in mice and mammalian in the MGI mine phenotype ontology database (http://www.mousemine.org/mousemine/begin.do).

CNVs founds in hiPSCs were also matched with gene locus which are known to be present in different human cancers by collecting information in TCGA consortium datasets on cBioPortal web application [55]. Workflow of bioinformatics pipeline is also drawn in Supplementary Figure 1.

## SUPPLEMENTARY MATERIALS FIGURES AND TABLES






