# Supplementary material for: Genomic landscape analyses of reprogrammed cells using integrative and non-integrative methods reveal variable cancer-associated alterations
Source: Oncotarget. 2019 Apr 12;10(28):2693–708. doi: 10.18632/oncotarget.26857 (PMC6505633; doi:10.18632/oncotarget.26857)
Supplement: Supplementary file 1 [file oncotarget-10-2693-s001.pdf]

Genomic landscape analyses of reprogrammed cells using integrative and non-integrative methods reveal variable cancer-associated alterations

SUPPLEMENTARY MATERIALS

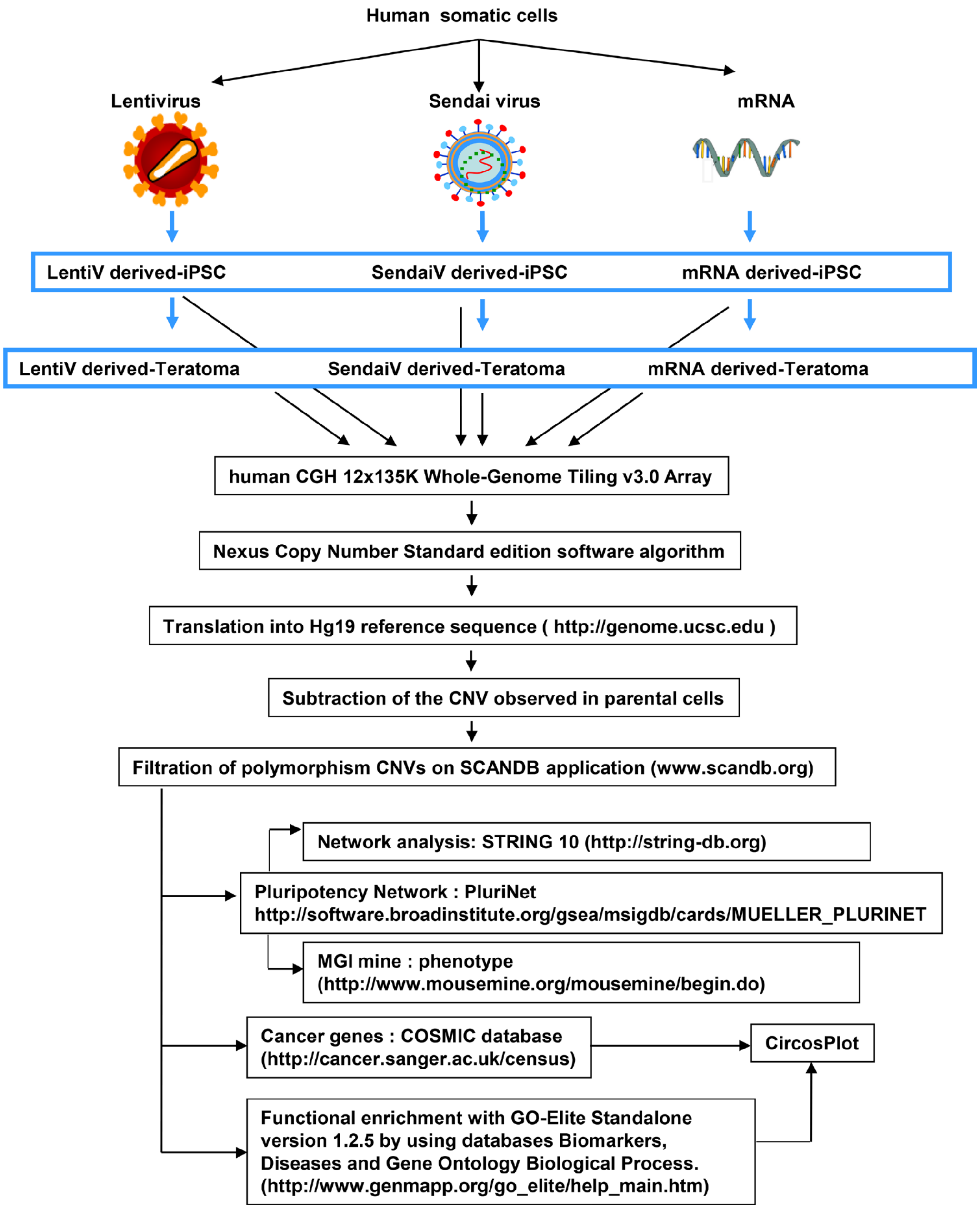

Supplementary Figure 1: Diagram of algorithm pipeline used during interpretation of aCGH experiments.

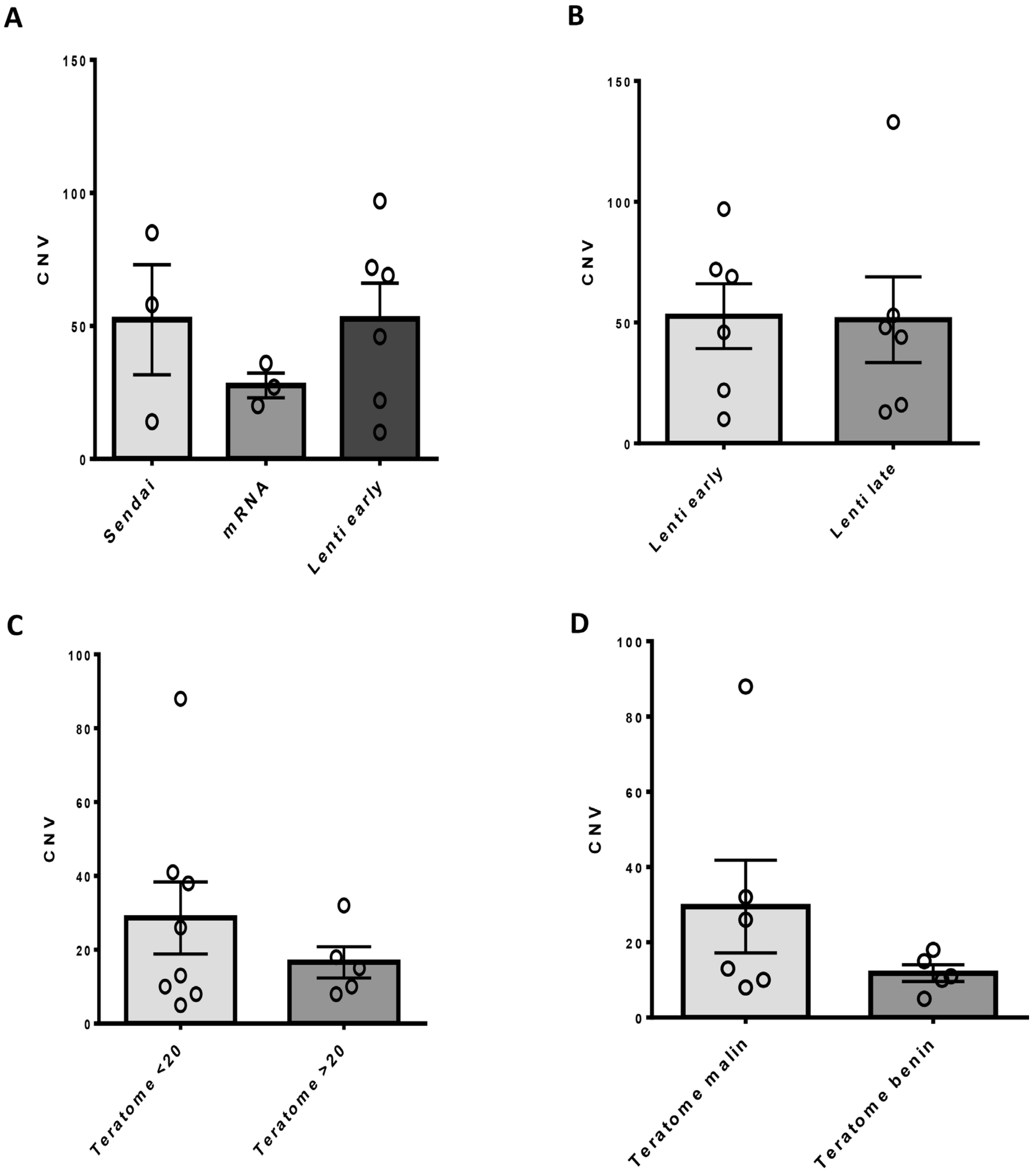

Supplementary Figure 2: Quantifications of Copy Number Variations (CNV) in each individual experiments of aCGH: (**A**) CNV quantification observed by aCGH in individual hiPSCs by 3 reprogramming methods: Sendai, mRNA, Lentivirus (early passage). (**B**) CNV quantification observed by aCGH in individual hiPSs taking in account early and late passages (threshold 20) for lentivirus method of hiPSCs reprograming. (**C**) CNV quantification observed by aCGH in individual teratoma taking in account early and late passages (threshold 20). (**D**) CNV quantification observed by aCGH in individual teratoma taking in account their malignant status.

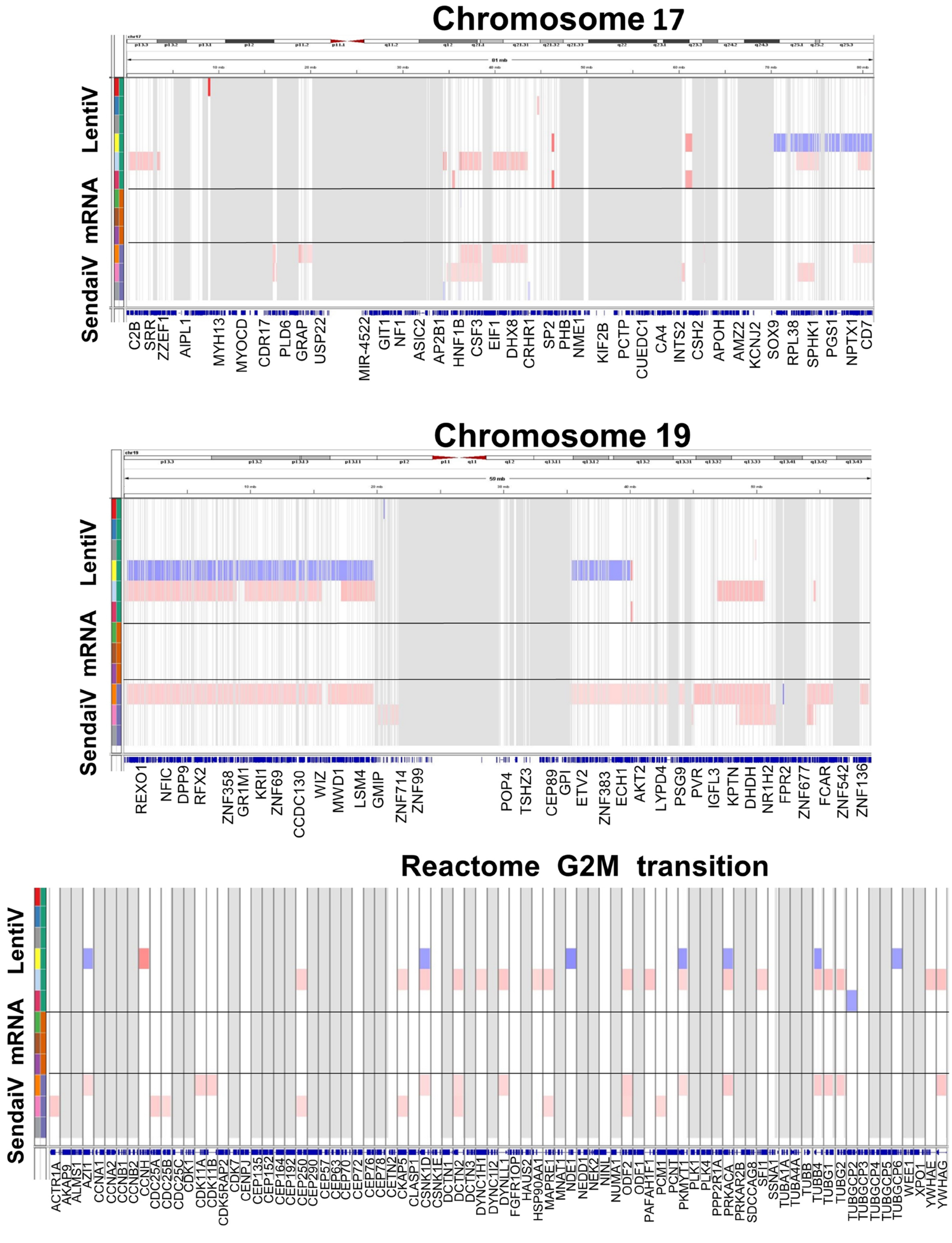

Supplementary Figure 3: Integrative genomic viewer analysis highlighting aCGH alterations: on chromosomes 17 and 19 and on cell cycle functionality (Reactome G2M transition) in hiPSCs cells experiments by the 3 different methods: lentivirus, Sendai virus and mRNA (gain in red, loss in blue).

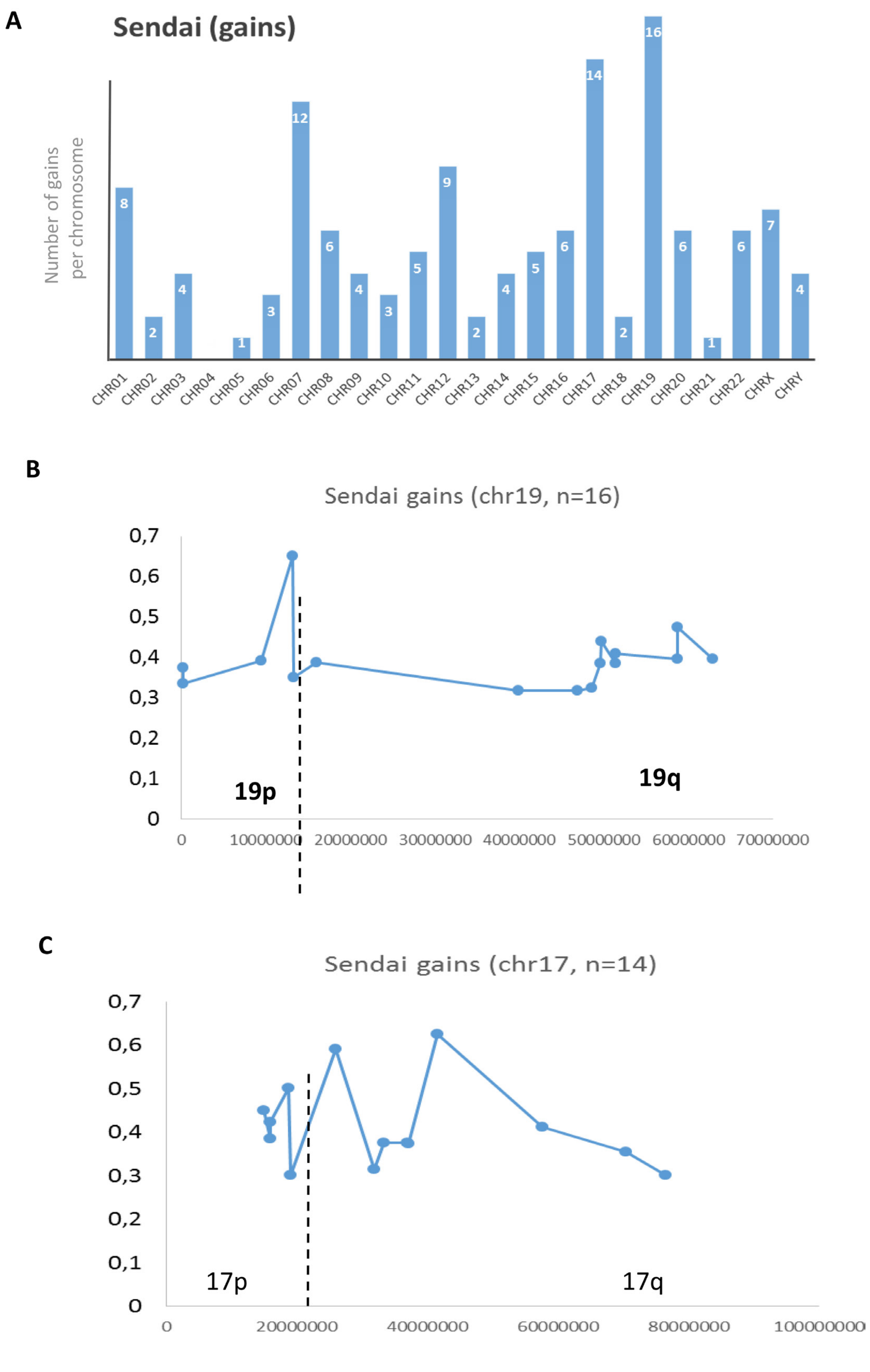

Supplementary Figure 4: Gain variations observed in IPSCs reprogrammed by Sendai virus are mainly observed on small chromosomes 17 and 19. (**A**) barplot of gain variation numbers on each chromosome of iPSCs reprogrammed with Sendai virus. (**B**) chromosome mapping (x-axis in pb) on chromosome 19 for the gain variation events observed in iPSCs reprogrammed by Sendai virus. (**C**) chromosome mapping (x-axis in pb) on chromosome 17 for the gain variation events observed in IPSCs reprogrammed by Sendai virus.

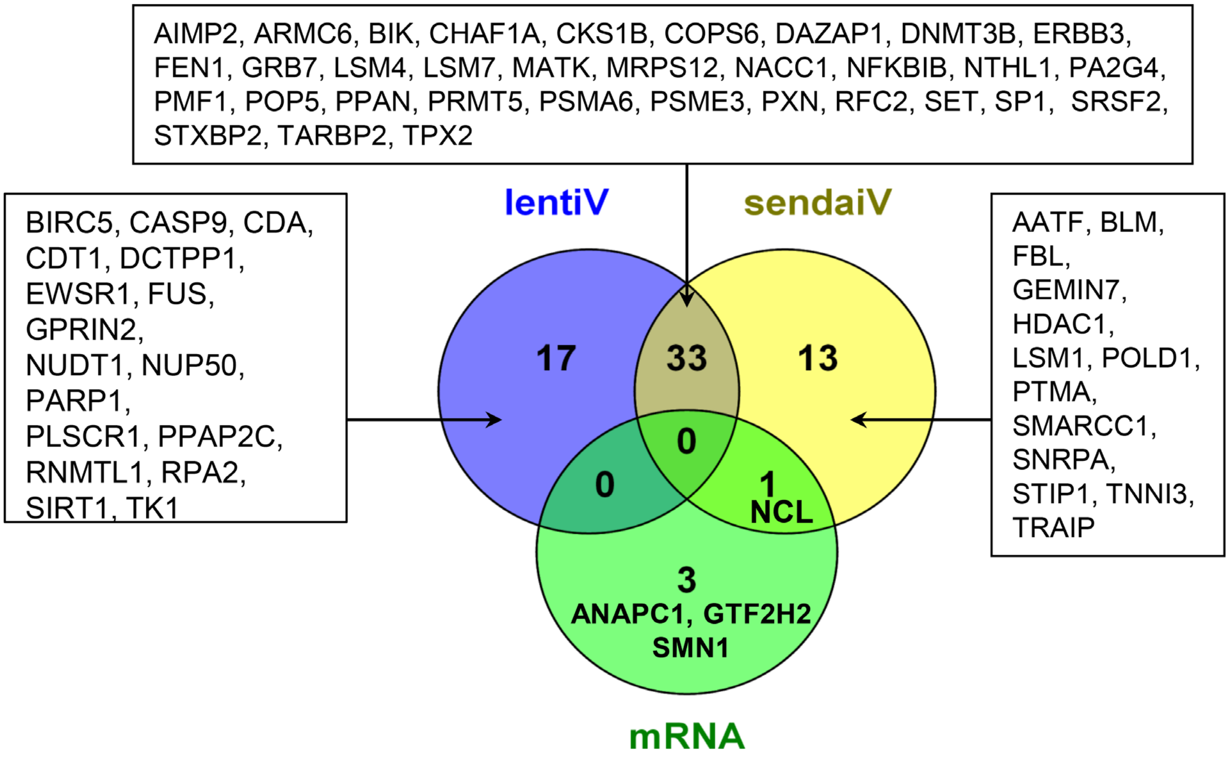

Supplementary Figure 5: Venn diagram comparing aCGH gene locus alterations related to PluriNet database for the hiPSCs cells by the 3 different reprogramming methods.

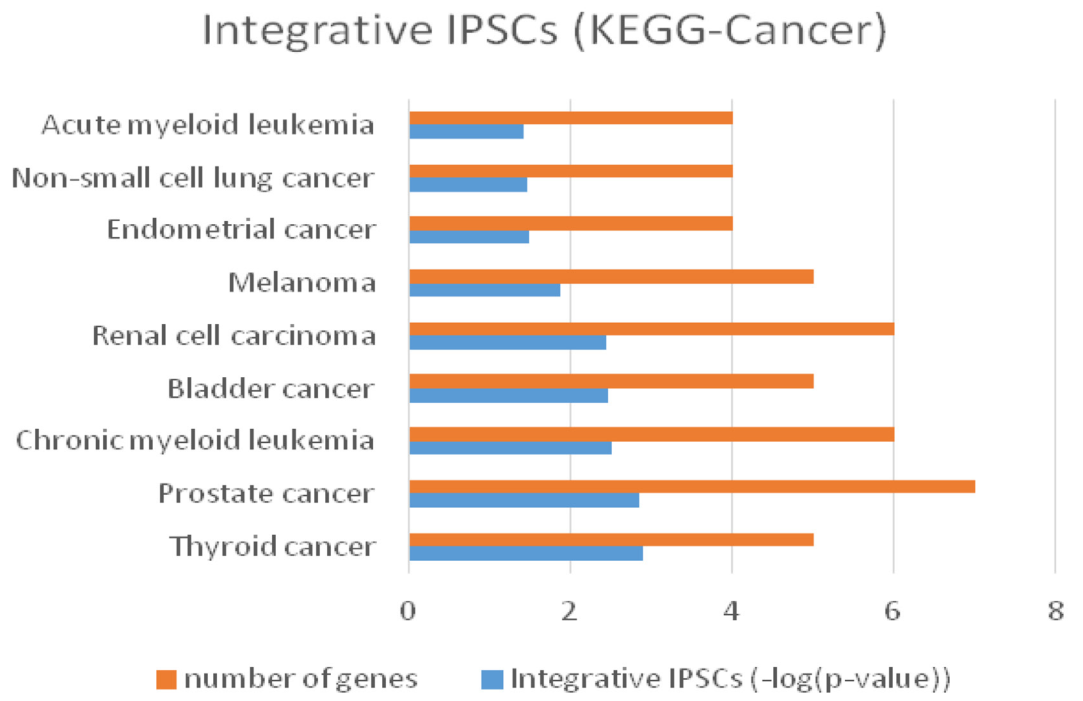

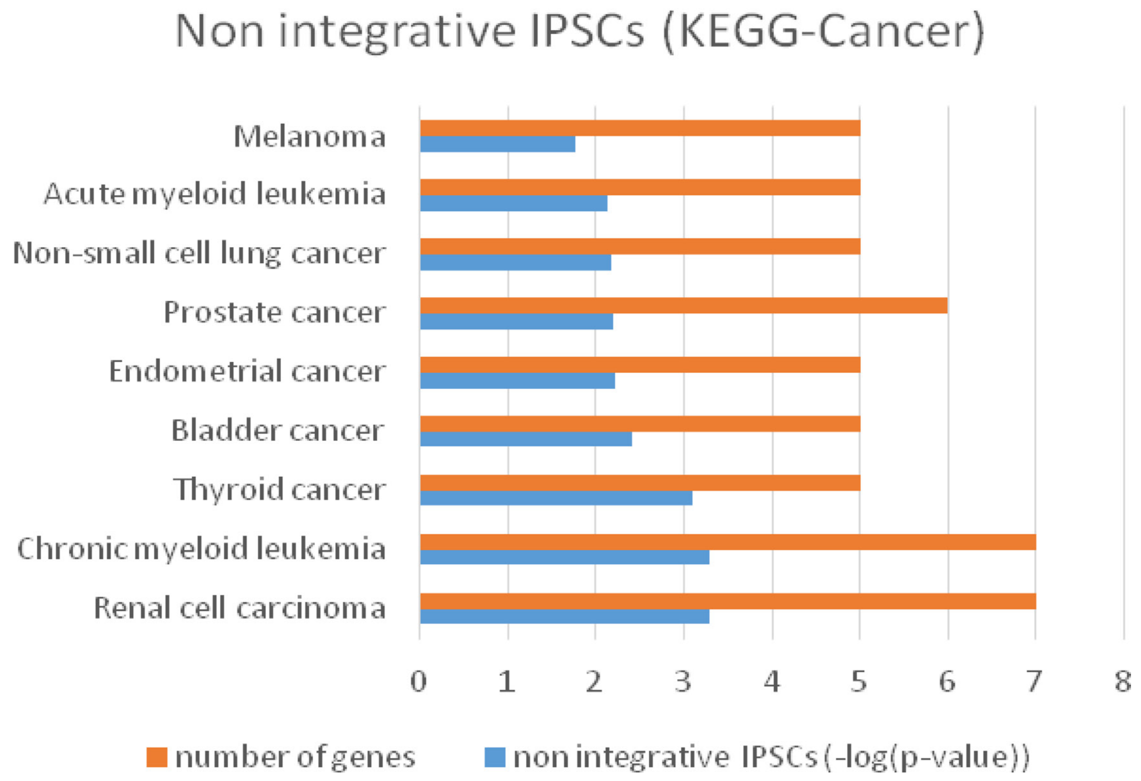

Supplementary Figure 6: Functional enrichment on Cancer-KEGG database of cancer related aCGH gene locus alterations for integrative or not integrative reprogramming methods of hiPSCs.

A

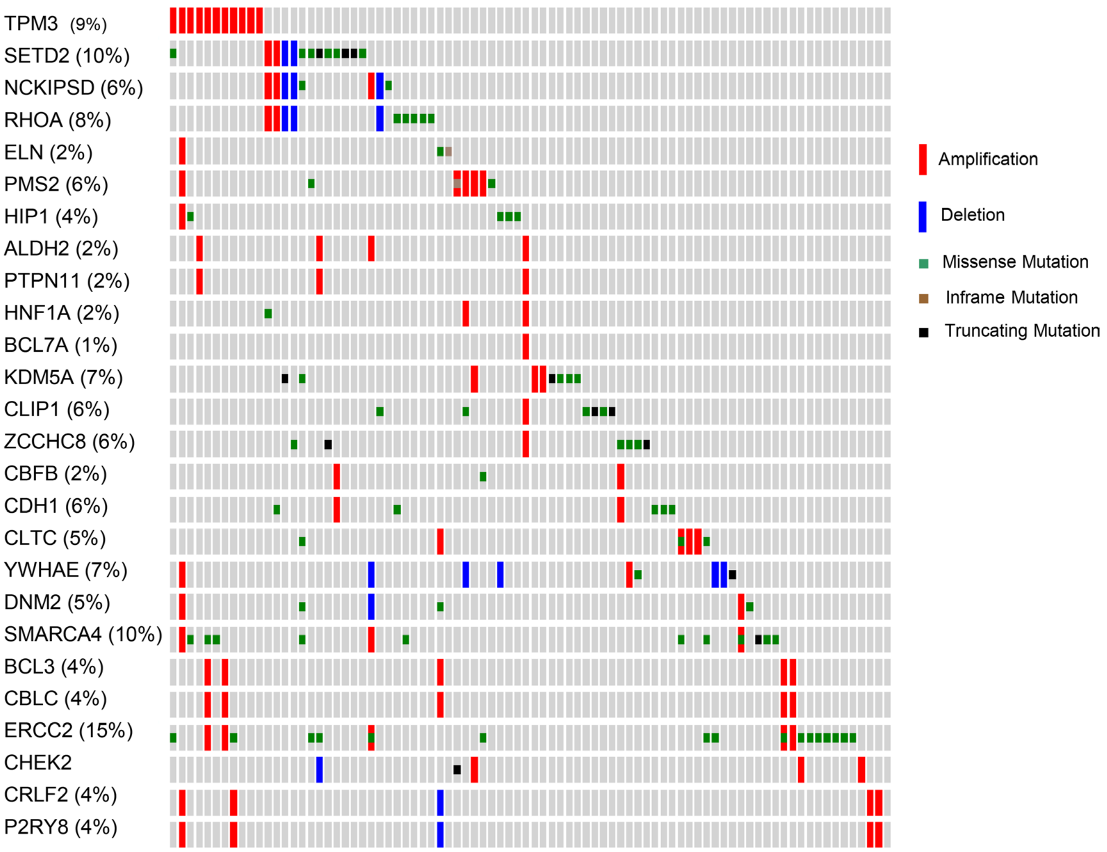

B

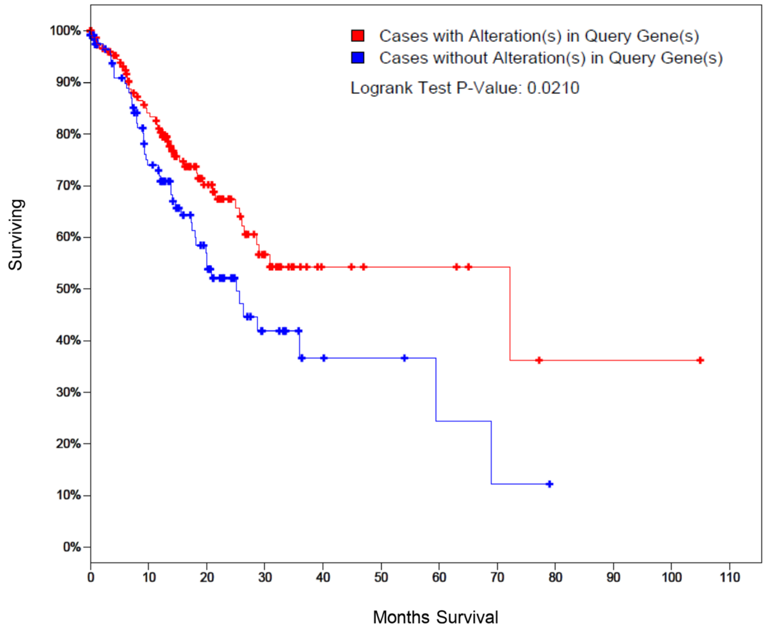

Supplementary Figure 7: (**A**) Oncoprint of alterations performed with genes found to be altered in teratoma with maligancy on TCGA dataset comprising samples of patient with stomach adenocarcinomas. (**B**) Kaplan Meier survival curve performed with genes found altered in teratoma with maligancy on TCGA dataset comprising samples of patient with stomach adenocarcinomas.

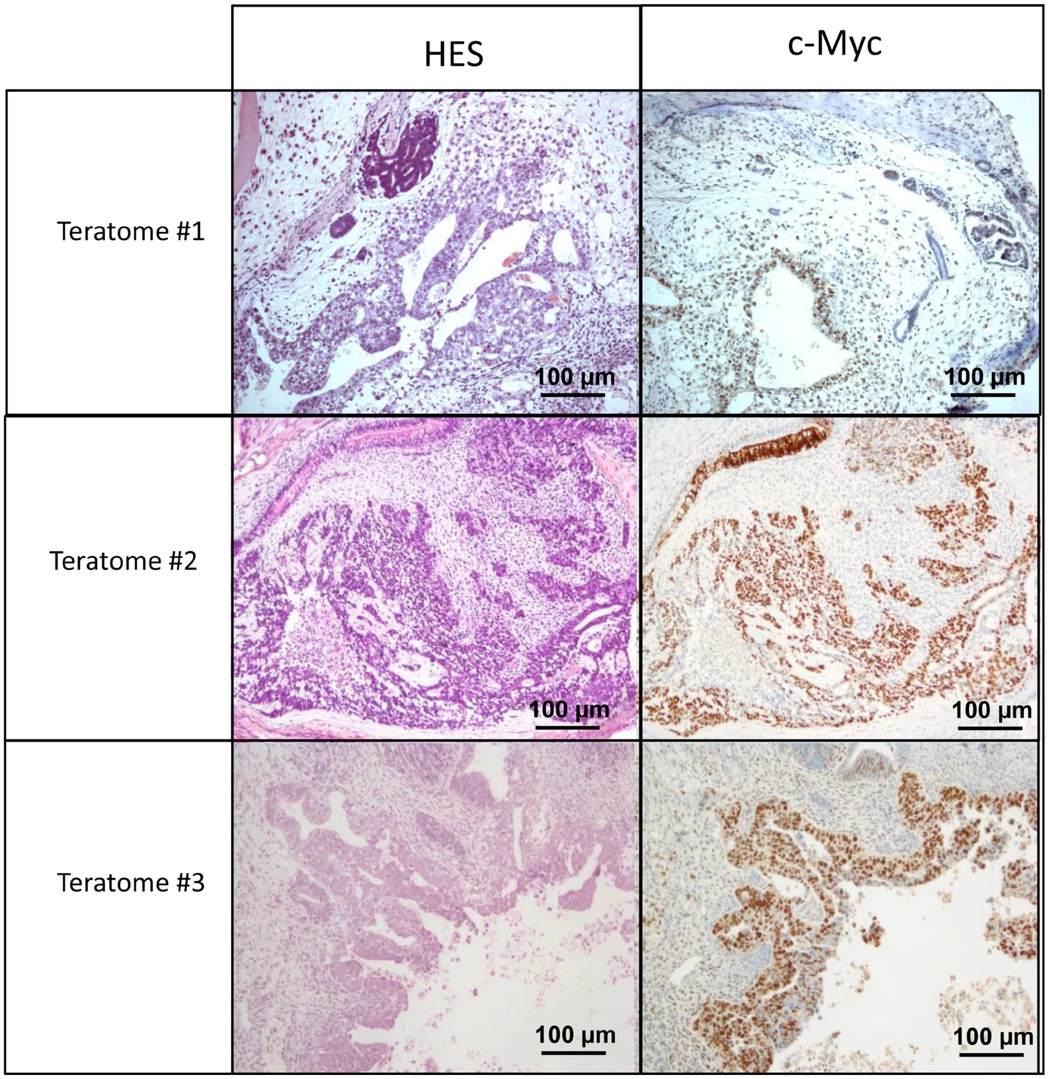

Supplementary Figure 8: Characteristics of iPS-derived teratomas with carcinoma positive for c-Myc. Carcinoma present in 3 different teratomas derived from Lentiviral-derived iPSCs stained with HES showing neoplastic cells positive c-Myc by immunohistochemistry staining.

Supplementary Table 1: Expression of transcriptional factors between 10 to 14 passages in individual iPSCs reprogrammed with lentiviral method

	Lin28	*Nanog*	*4-Oct*	*Sox2*
LV-1 Passage 13	NO	NO	YES	YES
LV-2 Passage 14	YES	NO	NO	NO
LV-3 Passage 14	NO	NO	YES	YES
LV-5 Passage 11	NO	YES	NO	NO
LV-5 Passage 10	NO	YES	NO	NO
LV-6 Passage 11	NO	YES	NO	NO
LV-1 Passage 32	NO	NO	NO	NO
LV-2 Passage 32	NO	NO	NO	NO
LV-3 Passage 22	NO	NO	NO	NO

Supplementary Table 2: Expression of transcriptional factors at early passage in individual iPSCs reprogrammed for Sendai virus method

	Klf4	*4-Oct*	*Sox2*	*cMyc*
SV-1 Passage 4	NO	NO	NO	NO
SV-2 Passage 3	NO	NO	NO	YES
SV-3 Passage 5	NO	NO	NO	NO
SV-1 Passage 10	NO	NO	NO	NO
SV-2 Passage 12	NO	NO	NO	NO
SV-3 Passage 10	NO	NO	NO	NO
SV-1 Passage 45	NO	NO	NO	NO
SV-2 Passage 45	NO	NO	NO	NO
SV-3 Passage 46	NO	NO	NO	NO

Supplementary Table 3: Functional enrichment of PluriNet related gene locus alteration detected by aCGH on hiPSCs cells reprogrammed by integrative method (lentivirus)

**a: GO Biological Process Database**			
**pathway ID**	**pathway description**	**count in gene set**	**false discovery rate**
GO:0006807	nitrogen compound metablic process	30	0.000374
GO:0034641	cellular nitrogen compound metabolic process	29	0.000374
GO:0044260	cellular macromolecule metabolic process	33	0.000374
GO:0043170	macromolecule metabolic process	34	0.000465
GO:0090304	nucleic acid metabolic process	25	0.000515
**b: GO Molecular Function Database**			
**pathway ID**	**pathway description**	**count in gene set**	**false discovery rate**
GO:0042802	identical protein binding	14	8.55e-5
GO:0003676	nucleic acid binding	24	0.000999
GO:0005515	protein binding	26	0.0016
GO:1901363	heterocyclic compound binding	27	0.0113
GO:0097159	organic cyclic compound binding	27	0.0117
**c: GO Cellular component Database**			
**pathway ID**	**pathway description**	**count in gene set**	**false discovery rate**
GO:0031981	nuclear lumen	27	2.9e-7
GO:0044428	nuclear part	28	2.9e-7
GO:0070013	intracellular organelle lumen	29	2.97e-7
GO:0043233	organelle lumen	29	3.24e-7
GO:0031974	membrane-enclosed lumen	29	3.58e-7
**d: KEGG Database**			
**pathway ID**	**pathway description**	**count in gene set**	**false discovery rate**
KEGG:03030	DNA replication	3	0.0122
KEGG:03410	Base excision repair	3	0.0122

Tables from a to d respectively represent enrichment performed on Gene-ontology biological process, Gene ontology molecular function, Gene ontology cellular component, KEGG pathways, results were represented by the number of genes enriched and by the adjust p-value by False discovery rate method, red node represent best biological process enriched.

Supplementary Table 4 : Functional enrichment of PluriNet related gene locus alteration detected by aCGH on hiPSCs cells reprogrammed by non-integrative method (Sendai virus)

**a: GO Biological Process Database**			
**Pathway ID**	**Pathway description**	**count in gene set**	**false discovery rate**
GO:0006396	RNA processing	13	6.51e-5
GO:0044260	Cellular macromolecule metabolic process	33	4.85e-3
GO:0000375	RNA splicing via transesterification reactions	7	1.48e-3
GO:0006397	mRNA processing	9	1.48e-3
GO:0010467	gene expression	24	1.48e-3
**b: GO Molecular Function Database**			
**Pathway ID**	**Pathway description**	**count in gene set**	**false discovery rate**
GO:0003676	nucleic acid binding	25	0.000421
GO:0003723	RNA binding	14	0.0146
GO:0097195	organic cyclic compound binding	27	0.0146
GO:1901363	heterocyclic compound binding	27	0.0146
GO:0047485	protein N terminus binding	4	0.0246
**c: GO Cellular Component Database**			
**Pathway ID**	**Pathway description**	**count in gene set**	**false discovery rate**
GO:0031981	nuclear lumen	32	1.56e-11
GO:0044428	nuclear part	32	9.09e-11
GO:0070013	intracellular organelle lumen	33	1.06e-10
GO:0043233	organelle lumen	33	1.24e-10
GO:0031974	membrane enclose lumen	33	1.46e-10
**d: KEGG pathways Database**			
**Pathway ID**	**Pathway description**	**count in gene set**	**false discovery rate**
KEGG:03030	DNA replication	3	0.0122
KEGG:03410	Base excision repair	3	0.0122
KEGG:03040	spliceosome	4	0.0171
KEGG:03420	nucleotide excision repair	3	0.0171
KEGG:03018	RNA degradation	3	0.0401

Tables from a to d respectively represent enrichment performed on Gene-ontology biological process, Gene ontology molecular function, Gene ontology cellular component, KEGG pathways, results were represented by the number of gene enriched and by the adjust *p*-value by the false discovery rate (FDR) method, red node represent best biological process enriched.

Supplementary Table 5: Functional enrichment performed on aCGH altered gene locus for the 3 different methods of reprogramming on hiPSCs cells. See Supplementary_Table_5

Supplementary Table 6: COSMIC database cancer related aCGH altered gene locus in hiPSCs cells with the 3 different methods of reprogramming

Samples	number of genes	gene set
LentiV derived-iPSCs	87	SPEN, MDS2, THRAP3, MLLT11, LMNA, PRCC, NTRK1, H3F3A, SDHB, SFPQ, CSF3R, ARNT, TPM3, MUC1, FANCD2, VHL, MITF, FOXP1, MECOM, RHOH, ELN, TRRAP, CARD11, PMS2, SBDS, HIP1, SET, ABL1, FNBP1, TET1, CREB3L1, DDB2, SDHAF2, HRAS, ERBB3, HNF1A, BCL7A, NACA, DDIT3, CDK4, CLIP1, ZCCHC8, HSP90AA1, TRAF7, IL21R, FUS, CBFB, CDH1, CREBBP, MYH11, CBFA2T3, MLLT6, LASP1, CDK12, ERBB2, RARA, RNF213, ASPSCR1, YWHAE, STAT5B, BRCA1, ETV4, H3F3B, SRSF2, CANT1, FSTL3, STK11, DNM2, SMARCA4, TPM4, CNOT3, TCF3, MAP2K2, SH3GL1, BRD4, JAK3, ELL, TFPT, ASXL1, EWSR1, NF2, EP300, MKL1, STAG2, ATP2B3, CRLF2, P2RY8
LentiV derived-teratoma	26	TPM3, SETD2, NCKIPSD, RHOA, ELN, PMS2, HIP1, ALDH2, PTPN11, HNF1A, BCL7A, KDM5A, CLIP1, ZCCHC8, CBFB, CDH1, CLTC, YWHAE, DNM2, SMARCA4, BCL3, CBLC, ERCC2, CHEK2, CRLF2, P2RY8
SendaiV derived-iPSCs	88	MLLT11, LMNA, PRCC, NTRK1, H3F3A, ARNT, TPM3, MUC1, FANCD2, VHL, SETD2, NCKIPSD, RHOA, MLLT4, ELN, TRRAP, PMS2, HIP1, PCM1, WHSC1L1, TCEA1, NCOA2, SET, ABL1, FNBP1, NFKB2, SUFU, DDB2, SDHAF2, MALAT1, HRAS, MEN1, DDX6, ERBB3, ALDH2, HNF1A, KDM5A, NACA, DDIT3, CDK4, CLIP1, ZCCHC8, POLE, CRTC3, BLM, IDH2, TRAF7, CBFB, CDH1, CREBBP, MLLT6, LASP1, CDK12, ERBB2, RARA, ASPSCR1, STAT5B, BRCA1, ETV4, H3F3B, SRSF2, FSTL3, STK11, DNM2, SMARCA4, TPM4, CIC, BCL3, CBLC, KLK2, ZNF331, CNOT3, TCF3, MAP2K2, SH3GL1, BRD4, JAK3, ELL, AKT2, ERCC2, TFPT, ASXL1, EP300, MKL1, GATA1, CRLF2, P2RY8, TFE3
SendaiV derived-teratoma	2	FUS, NF2
mRNA derived-iPSCs	1	ALK
mRNA derived-teratoma	0	0

Supplementary Table 7: Histological analysis of teratomas showing the proportion of neurectoderm, endoderm, mesoderm and malignant tissues found in teratoma generated with embryonic stem cells, integrative or non-integrative vectors

	hESC	Lentivirus	Sendai virus	mRNA
Neurectoderm				
Malpighian	7/8	15/21	17/19	2/2
Glial tissue	6/8	18/21	16/19	2/2
Neural crest	6/8	19/21	19/19	2/2
Endoderm				
Intestinal epithelium	8/8	21/21	19/19	2/2
Mesoderm				
Muscle	7/8	14/21	10/19	0/2
Cartilaginous	6/8	20/21	18/19	2/2
Bone	5/8	14/21	15/19	2/2
Fatty tissue	5/8	8/21	4/19	2/2
Malignant tissue	0/8	8/21	0/19	0/2

Supplementary Table 8: Functional enrichment performed on aCGH altered gene loci for hiPSCs cells taking into account the number of passage and also on teratoma taking into account malignancy status and number of passages. See Supplementary_ Table_8

Supplementary Table 9: COSMIC database cancer related aCGH altered gene loci in hiPSCs and teratomas generated with lentiviral vectors, taking into account the number of passage (less or more than 20 passages) and the malignancy status of teratoma (with or without malignancy)

Samples	number of genes	gene set
iPSCs ($p < 20$) $n = 6$	89	ABL1, ARNT, ASPSCR1, ASXL1, ATP2B3, BCL7A, BRCA1, BRD4, CANT1, CARD11, CBFA2T3, CBFB, CDH1, CDK12, CDK4, CLIP1, CNOT3, CREB3L1, CREBBP, CRLF2, CSF3R, DDB2, DDIT3, DNM2, ELL, ELN, EP300, ERBB2, ERBB3, ETV4, EWSR1, FANCD2, FNBP1, FOXP1, FSTL3, FUS, H3F3A, H3F3B, HIP1, HNF1A, HRAS, HSP90AA1, IL21R, JAK3, LASP1, LMNA, MAP2K2, MDS2, MECOM, MITF, MKL1, MLLT11, MLLT6, MUC1, MYH11, NACA, NF2, NTRK1, P2RY8, PMS2, PRCC, RARA, RHOH, RNF213, SBDS, SDHAF2, SDHB, SEPT5, SEPT9, SET, SFPQ, SH3GL1, SMARCA4, SPEN, SRSF2, STAG2, STAT5B, STK11, TCF3, TET1, TFPT, THRAP3, TPM3, TPM4, TRAF7, TRRAP, VHL, YWHAE, ZCCHC8
iPSCs ($p > 20$) $n = 6$	76	ALDH2, ASPSCR1, ASXL1, ATP2B3, BCL11A, BCL11B, BCL3, BCL7A, BRD3, CAMTA1, CANT1, CARD11, CASC5, CBFA2T3, CBLB, CBLC, CCNE1, CDC73, CDH1, CDK12, CLIP1, CRLF2, ELN, ERBB2, ERBB3, ERCC2, ERG, FANCF, FGFR1OP, FOXP1, HERPUD1, HIP1, HNF1A, IKZF1, LASP1, LMNA, MECOM, MITF, MLLT4, MLLT6, MUC1, NACA, NCKIPSD, NR4A3, NTRK1, OLIG2, P2RY8, PML, PMS2, POLE, PRCC, PTPR, RAD51B, RALGDS, RARA, RHOA, RNF213, RPL22, RUNX1, SH2B3, SS18L1, STAG2, TERT, TET1, TET2, TFG, TFPT, TMPRSS2, TNFRSF14, TPM3, TRRAP, U2AF1, XPC, YWHAE, ZCCHC8, ZNF331
Teratome $p < 20$ $n = 8$	55	TPM3, ELK4, RAF1, SETD2, NCKIPSD, RHOA, RHOH, ELN, PMS2, HIP1, TET1, ALDH2, PTPN11, HNF1A, BCL7A, KDM5A, NACA, CLIP1, ZCCHC8, CBFB, CDH1, MLLT6, LASP1, CDK12, ERBB2, RARA, CLTC, YWHAE, STAT5B, BRCA1, ETV4, FSTL3, STK11, DNM2, SMARCA4, TPM4, BCL3, CBLC, KLK2, ZNF331, TCF3, MAP2K2, SH3GL1, BRD4, JAK3, ELL, ERCC2, OLIG2, RUNX1, ERG, TMPRSS2, U2AF1, CHEK2, CRLF2, P2RY8
Teratome $p > 20$ $n = 3$	1	FLI1
Teratoma with malignancy $n = 8$	54	TPM3, SETD2, NCKIPSD, RHOA, RHOH, ELN, TRRAP, PMS2, HIP1, FLI1, ALDH2, PTPN11, HNF1A, BCL7A, KDM5A, CLIP1, ZCCHC8, CBFB, CDH1, MLLT6, LASP1, CDK12, ERBB2, RARA, CLTC, YWHAE, STAT5B, BRCA1, ETV4, FSTL3, STK11, DNM2, SMARCA4, TPM4, BCL3, CBLC, KLK2, ZNF331, TCF3, MAP2K2, SH3GL1, BRD4, JAK3, ELL, ERCC2, OLIG2, RUNX1, ERG, TMPRSS2, U2AF1, CHEK2, STAG2, CRLF2, P2RY8
Teratoma without malignancy $n = 7$	8	ELK4, RAF1, RHOH, PMS2, TET1, NACA, CDK12, ELL

Supplementary Table 10: Characteristics of 45 hiPSCs: cell type

iPSCs	Cell of origin	Factors	SSEA4 (%)	Tra-1-60 (%)	Karyoytpe
LV-1	Human Amniocyte	LV OSLN	99,1	ND	46,XY
LV-2	Human Amniocyte	LV OSLN	99,79	ND	46,XY
LV-3	Human Amniocyte	LV OSLN	99,11	ND	ND
LV-4	Human Amniocyte	LV OSLN	99,77	ND	ND
LV-5	Human Amniocyte	LV OSLN	99,22	ND	ND
LV-6	Human Amniocyte	LV OSLN	98,08	66,32	46, XX
LV-7	Human Amniocyte	LV OSLN	99,3	ND	ND
LV-8	Human Amniocyte	LV OSLN	99,11	ND	ND
LV-9	Human Amniocyte	LV OSLN	99,91	84,01	46,XY
LV-10	Human Amniocyte	LV OSLN	99,22	ND	ND
LV-11	Human Amniocyte	LV OSLN	47,4	25,86	46,XY
LV-12	Human Amniocyte	LV OSLN	98,78	98,69	46, XY
LV-13	Human Amniocyte	LV OSLN	99,6	92,3	46, XY
LV-14	Human Amniocyte	LV OSLN	98,31	94,47	46, XY
LV-15	Human Amniocyte	LV OSLN	98,59	88,7	46,XY
LV-16	Human Amniocyte	LV OSLN	98,49	78,8	46,XY
LV-17	Human Amniocyte	LV OSLN	99,8	91,38	46,XY
LV-18	Human Amniocyte	LV OSLN	99,8	91,38	46,XY
LV-19	Fibroblasts	LV OSKC	ND	ND	46, XX
LV-20	Fibroblasts	LV OSKC	97,8	97,6	46, XY
LV-21	Fibroblasts	LV OSKC	99,17	97,31	46, XX
SV-1	CD34	SV OSKC	68,25	8,86	46, XY
SV-2	CD34	SV OSKC	100	99,87	46, XY
SV-3	PBML	SV OSKC	98,31	97,97	46, XX
SV-4	Fibroblasts	SV OSKC	98,5	94,17	46, XY
SV-5	PBMC	SV OSKC	99,9	99,03	46, XX
SV-6	PBMC	SV OSKC	99,7	99,19	46, XX
SV-7	PBMC	SV OSKC	99,3	78,55	46, XY
SV-8	PBMC	SV OSKC	89,65	86,2	46, XY
SV-9	PBMC	SV OSKC	99,39	65,3	46, XY
SV-10	PBMC	SV OSKC	99,52	93,06	46, XY
SV-11	CD34	SV OSKC	99,65	97,93	46, XY
SV-12	fibroblasts	SV OSKC	99,81	96,09	46, XY
SV-13	PBMC	SV OSKC	99,96	96,9	46, XX
SV-14	PBMC	SV OSKC	97,59	94,78	46, XY
SV-15	fibroblasts	SV OSKC	82,61	75,59	46, XY
SV-16	Fibroblasts	SV OSKC	97,6	83,54	46, XY
SV-17	Fibroblasts	SV OSKC	98,5	94,17	46, XY
SV-18	MSC	SV OSKC	99,17	96,44	46, XY
SV-19	Fibroblasts	SV OSKC	98,2	96,5	46, XY
mRNA-1	Fibroblasts	mRNA OSKM	96,2	85,6	46, XY
mRNA-2	Fibroblasts	mRNA OSKM	95,9	94,2	46, XY
