# Supplementary material for: Genomic landscape analyses of reprogrammed cells using integrative and non-integrative methods reveal variable cancer-associated alterations
Source: Oncotarget. 2019 Apr 12;10(28):2693–708. doi: 10.18632/oncotarget.26857 (PMC6505633; doi:10.18632/oncotarget.26857)
Supplement: Supplementary file 2 [file oncotarget-10-2693-s002.doc]

**Supplementary Table 5: Functional enrichment performed on aCGH altered gene locus for the 3 different methods of reprogramming on hiPSCs cells**

|  |  |  |  |  |  |
| --- | --- | --- | --- | --- | --- |
| LentiV-  derived  iPSCs  (p<20) | Biomarkers | iPSCs  specific  markers | 20 | ANKRD11, ATP6V0A2, BRD4, CARM1, CCDC94, DBNDD1, GABRA3, GLTSCR1, GRIN2D, LSM12, MRPS34, OLFM2, PLA2G3, PNKP, POU6F2, RBM11, SETD1A, TAF6, TMEM160, VKORC1L1 | p<0.001 |
|  | Gene Ontology  Biological  Process | Chromatin  silencing | 11 | BAZ2A, DNMT3B, HDAC5, MBD3, SIRT1, SIRT2, SIRT4, SIRT6, SIRT7, SMARCA4, UBTF | p<0.001 |
|  | Gene Ontology  Biological  Process | Generation of  female  gametes | 13 | CGB, CGB1, CGB5, DYNLL1, FSHR, HORMAD1, MSH4, NANOS3, NOBOX, PAQR7, PARN, SRC, TOB2 | p<0.001 |
|  | Gene Ontology  Biological  Process | Lipid  metabolism | 15 | A4GALT, ARSA, B4GALNT1,  BAX, CERK, CLN3, ENPP7,  GAL3ST1, GBA, LARGE,  PSAP, SMPD3, ST6GALNAC3,  ST6GALNAC5, ST6GALNAC6 | P=0.045 |
|  | Gene Ontology  Biological  Process | Response  to glucagon | 13 | ADCY8, ADCY9, ASL, ASS1,  GNB2, GNG11, GNG13,  GNG3, GNG7, GNG8,  PRKACA, PRKACB, PRKAR1B | p<0.001 |
|  | Gene Ontology  Biological  Process | Mitochondria  organization | 12 | BAX, BBC3, BCL2L1, BNIP3, CLN8, HSP90AA1, SAMM50, SNCA, TAZ, TIMM13, TIMM22, TIMM50 | p<0.001 |
|  | Gene Ontology  Biological  Process | Plama  membrane | 11 | A4GALT, CALCR, CRB3, P2RX7, PLSCR1, PLSCR2, PLSCR4, RAMP2, SYT7, TRIM72, ZFYVE27 | p<0.001 |
|  | Gene Ontology  Biological  Process | Retrograde  transport  to the golgi | 10 | BET1L, FAM109B, FAM21B, FAM21C, SNX1, SNX6, SNX8, STX10, STX5, VPS26A | p<0.001 |
|  | Gene Ontology  Biological  Process | Telomere  maintenance | 3 | ACD, TERF2, TERF2IP | P=0.0025 |
|  |  |  |  |  |  |
| SendaiV-  derived  iPSCs  (p<20) | Biomarkers | iPSCs  specific  markers | 15 | ATP6V0A2, BRD4, CARM1, CCDC94, CNFN, CYP2S1, GLTSCR1, GRIN2D,  LSM12, MESP2, MRPS34, OLFM2, PNKP, TAF6, TMEM160 | p<0.001 |
|  | Gene Ontology  Biological  Process | Viral cycle | 26 | FAU, RPL12, RPL13, RPL13A, RPL18A, RPL19,  RPL23, RPL27, RPL28, RPL35, RPL3L, RPL41,  RPLP0, RPLP2, RPS11, RPS15, RPS16, RPS2,  RPS25, RPS26, RPS27, RPS28, RPS9, SLC25A6,  TGFB1, UBA52 | p=0.0185 |
|  | Gene Ontology  Biological  Process | Viral transcription | 25 | FAU, RPL12, RPL13, RPL13A, RPL18A, RPL19,  RPL23, RPL27, RPL28, RPL35, RPL3L, RPL41,  RPLP0, RPLP2, RPS11, RPS15, RPS16, RPS2,  RPS25, RPS26, RPS27, RPS28, RPS9, UBA52,  USF2 | p=0.005 |
|  | Gene Ontology  Biological  Process | Positive  regulation of  viral reproduction | 22 | CCL4, CDK9, ELL, EP300, ERCC2, GTF2F1, GTF2H3, HDAC1, HPN, POLR2E, POLR2G, POLR2J, POLR2L, RAD23A, SMARCA4, SP1, SUPT5H, TARBP2, TCEA1, TCEB2, TFAP4, TOP2A | p=0.008 |
|  | Gene Ontology  Biological  Process | Histone  methylation | 17 | ASH2L, BAZ2A, CARM1, DOT1L, EZH1, MEN1, MLL, MLL4, PRMT1, PRMT5, PRMT7, SETD1B, SETD2, SETD8, SETDB1, SUV420H2, WHSC1L1 | p<0.001 |
|  |  |  |  |  |  |
| mRNA-  derived  iPSCs  (p<20) | Biomarkers | iPSCs specific  markers | 0 | 0 | - |
|  | Gene Ontology  Biological  Process | Gene expression | 8 | CNOT8, GEMIN5, GTF2H2, LTB, POLR2F, POLR2J, SMN1, SMN2 | p=0.0065 |
|  | Gene Ontology  Biological  Process | Embryo  development | 6 | APBA2, FOXD4L3, FOXD4L4, FOXD4L6, SOX10, TPI1 | p=0.0045 |

Enrichment were performed on Biomart-biomarkers and Gene Ontology-biological process databases, number of genes enriched are presented with the corrected *p*-value of enrichment after 2000 permutations.
