# Supplementary material for: Genomic landscape analyses of reprogrammed cells using integrative and non-integrative methods reveal variable cancer-associated alterations
Source: Oncotarget. 2019 Apr 12;10(28):2693–708. doi: 10.18632/oncotarget.26857 (PMC6505633; doi:10.18632/oncotarget.26857)
Supplement: Supplementary file 3 [file oncotarget-10-2693-s003.doc]

**Supplementary Table 8: Functional enrichment performed on aCGH altered gene locus for hiPSCs cells**

|  |  |  |  |  |  |
| --- | --- | --- | --- | --- | --- |
| **Samples** | **database** | **gene set** | **number  of gene** | **genes** | ***P* value** |
| iPSCs  (p<20) n=6 | Biomarkers | iPSCs specific  markers | 20 | ANKRD11, ATP6V0A2,  BRD4, CARM1, CCDC94,  DBNDD1, GABRA3,  GLTSCR1, GRIN2D,  LSM12, MRPS34,  OLFM2, PLA2G3,  PNKP, POU6F2, RBM11,  SETD1A, TAF6,  TMEM160, VKORC1L1 | <0.0001 |
|  | Diseases | Bile Duct  Neoplasms | 7 | FSCN1, MGMT, MKI67,  MSLN, MUC1, MUC16,  RUNX3 | 0.003 |
|  | Diseases | Neuroendocrines  tumors | 10 | ASS1, CDK4, CYP2E1,  MC1R, MGMT, NFKBIA,  SDHB, SNCA, VHL,  XRCC3 | <0.0001 |
|  | Diseases | Nevi and melanomas | 6 | ASS1, CDK4, MC1R,  MGMT, NFKBIA,  XRCC3 | 0.003 |
|  | Gene Ontology- Biological Process | MAPK signaling | 33 | ADAM8, AVPI1, C5AR1,  DRD4, EFNA1, FCER1A,  FGF10, GNG3, IKBKG,  IQGAP3, IRAK1, IRAK2,  ITGA1, MADD, MAP2K2,  MAP2K7, MAP3K6,  MAP3K7, MAP4K1,  MAPK1, MAPK11,  MAPK3, MAPK8IP3,  P2RX7, PDE6G,  PKN1, PLA2G1B,  PRDX2, PTPLAD1,  PXN, SHC1, UBA52,  WNT7B | 0.0375 |
|  | Gene Ontology Biological Process | Chromatine  silencing | 11 | BAZ2A, DNMT3B, HDAC5,  MBD3, SIRT1, SIRT2,  SIRT4, SIRT6,  SIRT7, SMARCA4,  UBTF | 0.0001 |
| iPSCs  (p>20) n=6 | Biomarkers | Embryo | 13 | AMBN, DSC1, KRT1,  KRT4, KRT6A, KRT76,  MATN1, PSAPL1,  SERPINB12, SERPINB7,  TMPRSS11F, UGT2A1,  ZCCHC5 | 0.0001 |
|  | Diseases | Carcinoma, Islet  Cell | 3 | BTC, MGMT, SSTR4 | 0.019 |
| Teratoma  (p<20) n=8 | Biomarkers | IPSCs specific  markers | 11 | ATP6V0A2, BRD4,  CARM1, CCDC94,  GLTSCR1, GRIN2D,  LSM12, OLFM2,  PNKP, SETD1A,  TMEM160 | <0.001 |
|  | Biomarkers | Embryoid  Body  Day 4 | 7 | ETV4, KLK10, NANOG,  NUTF2, QTRT1,  RLN3, SUMF2 | 0.002 |
|  | Gene Ontology  Biological  Process | mRNA splicing | 37 | ARL6IP4, CASC3, CCAR1,  CD2BP2, DDX46, DHX8,  DNAJC8, EFTUD2,  GEMIN4, GEMIN7,  GTF2F1, LSM4,  LSM7, NOL3, POLR2E,  POLR2G, POLR2J,  PPAN, PRPF8,  PTBP1, RBMY1A1,  RBMY1B, RBMY1D,  RBMY1E, RBMY1F,  RBMY1J, SF3B3,  SMN1, SMN2, SNRPD1,  SNRPD2, SRRM1,  TSEN2, U2AF1,  UPF3B, XAB2, ZCCHC8 | 0.02 |
|  | Gene Ontology  Biological  Process | mRNA  catabolism | 20 | CASC3, ETF1, RPL13A,  RPL18A, RPL19, RPL23,  RPL27, RPL39, RPL6,  RPL9, RPLP0, RPS11,  RPS15, RPS15A,  RPS27, RPS28, SMG1,  UBA52, UPF1, UPF3B | 0.042 |
|  | Gene Ontology Biological  Process | Nucleotide  Excision  Repair | 14 | ERCC1, ERCC2,  GTF2H2, GTF2H3,  LIG1, POLD1, POLE2,  POLR2E, POLR2G,  POLR2J, RFC1,  RFC2, RFC5, XAB2 | 0.001 |
|  | Gene Ontology Biological  Process | Viral  transcription | 15 | RPL13A, RPL18A, RPL19,  RPL23, RPL27, RPL39,  RPL6, RPL9, RPLP0,  RPS11, RPS15, RPS15A,  RPS27, RPS28, UBA52 | 0.049 |
|  | Gene Ontology  Biological  Process | DNA replication | 12 | BAZ1A, CDK2AP1, LIG1,  PNKP, POLD1, POLE2,  PRIM1, RFC1, RFC2,  RFC5, TK2, TOP2A | <0.001 |
|  | Gene Ontology Biological  Process | Protein translation | 16 | ETF1, RPL13A, RPL18A,  RPL19, RPL23, RPL27,  RPL39, RPL6, RPL9,  RPLP0, RPS11, RPS15,  RPS15A, RPS27, RPS28,  UBA52 | 0.0375 |
| Teratoma  (p>20) n=5 | Gene Ontology  Biological  Process | Spermato -genesis | 20 | ADCYAP1R1, BCL2L10,  BPY2, BPY2B, BPY2C,  CDY1, CDY1B, CDY2A,  CDY2B, DAZ1, DAZ2,  DAZ3, DAZ4, RBMY1A1,  RBMY1B, RBMY1F, RBMY1J, TSPY1,  TSPY2, UBE2B | <0.001 |
|  | Gene Ontology  Biological  Process | Fertilization | 5 | BPY2, BPY2B, BPY2C,  DAZ2, DAZ4 | <0.001 |
|  | Gene Ontology  Biological  Process | mRNA splicing | 8 | DDX46, PPP2CA,  RBMY1A1, RBMY1B,  RBMY1D, RBMY1E,  RBMY1F, RBMY1J | <0.001 |
|  | Gene Ontology  Biological  Process | mRNA processing | 8 | DDX46, GTF2H2,  RBMY1A1, RBMY1B,  RBMY1D, RBMY1E,  RBMY1F, RBMY1J | <0.001 |
| Teratoma  with  malignancy n=8 | Biomarkers | IPSCs specific  markers | 23 | AIRE, ATP6V0A2, BRD4,  CARM1, CHRNA9, CCDC94,  DPPA2, DPPA4, DNMT3L,  EMID2, ETV4, GDF3,  GLTSCR1, GRIN2D,  NANOG, LSM12, OLFM2,  PNKP, RNASEH2A,  SETD1A, TAF6, TMEM160,  ZIC3 | <0.001 |
|  | Biomarkers | EB markers  at day 4 | 7 | ETV4, KLK10, NANOG,  NUTF2, QTRT1, RLN3,  SUMF2 | 0.002 |
|  | Gene Ontology  Biological  Process | DNA replication | 28 | ACHE, BAZ1A, BRCA1,  CDC25A, CDC6,  CDK2AP1, CHAF1A,  CHAF1B, KCTD13,  LIG1, MCM3AP,  NFIC, NFIX, PNKP,  POLD1, RAD17,  RAD9B, RFC1,  RFC2, RFC5,  RNASEH2A,  RUVBL2, TERF2,  TERF2IP, TK2, TOP2A,  TREX1, UPF1 | 0.016 |
|  | Gene Ontology  Biological  Process | Initiation of  transcription | 19 | APITD1, ERCC2, GTF2F1,  GTF2H2, GTF2H3, GTF2I,  MAPK3, MAZ, POLR2E,  POLR2G, POLR2J,  POLRMT, PTRF, RTF1,  TAF12, TAF6, TAF6L,  TAF9, UBTF | 0.001 |
|  | Gene Ontology  Biological  Process | Nucleotide  Excision  Repair | 13 | ERCC1, ERCC2,  GTF2H2, GTF2H3,  LIG1, POLD1, POLR2E,  POLR2G, POLR2J,  RFC1, RFC2, RFC5,  XAB2 | <0.001 |
|  | Gene Ontology  Biological  Process | Protein  elongation | 18 | EEF1A1, EEF1B2,  RPL13A, RPL18A,  RPL19, RPL23, RPL27,  RPL39, RPL6, RPL9,  RPLP0, RPS11, RPS15,  RPS15A, RPS27, RPS28,  TUFM, UBA52 | 0.028 |
|  | Gene Ontology  Biological  Process | Protein termination | 16 | ETF1, RPL13A, RPL18A,  RPL19, RPL23, RPL27,  RPL39, RPL6, RPL9,  RPLP0, RPS11, RPS15,  RPS15A, RPS27, RPS28,  UBA52 | 0.039 |
| Teratoma  without  malignancy n=7 | Biomarkers | Pluripotent  stem cell  markers | 3 | CHRNA9, GDF3, NANOG | 0.03 |
|  | Gene Ontology  Biological  Process | Spermato- genesis | 22 | BPY2, BPY2B, BPY2C,  CCNB1, CDY1, CDY1B,  CDY2A, CDY2B, DAZ1,  DAZ2, DAZ3, DAZ4,  RBMY1A1, RBMY1B,  RBMY1F, RBMY1J,  SLC2A14, SPAG9,  TSPY1, TSPY2,  UBE2B, USP42 | <0.001 |
|  | Gene Ontology  Biological  Process | Fertilization | 5 | BPY2, BPY2B, BPY2C,  DAZ2, DAZ4 | <0.001 |
|  | Gene Ontology  Biological  Process | mRNA processing | 13 | APOBEC1, CCAR1,  DDX46, GTF2H2,  RBMY1A1, RBMY1B,  RBMY1D, RBMY1E,  RBMY1F, RBMY1J,  SMN1, SMN2, TSEN2 | <0.001 |
|  | Gene Ontology  Biological  Process | mRNA splicing | 12 | CCAR1, DDX46, PPP2CA,  RBMY1A1, RBMY1B,  RBMY1D, RBMY1E,  RBMY1F, RBMY1J,  SMN1, SMN2, TSEN2 | <0.001 |
|  | Gene Ontology  Biological  Process | Mesoderm  development | 4 | GDF3, PPP2CA, TSPY1,  TSPY2 | 0.0025 |
|  | Gene Ontology  Biological  Process | mRNA splicing | 12 | CCAR1, DDX46, PPP2CA,  RBMY1A1, RBMY1B,  RBMY1D, RBMY1E,  RBMY1F, RBMY1J,  SMN1, SMN2, TSEN2 | <0.001 |
|  | Gene Ontology  Biological  Process | Mesoderm  development | 4 | GDF3, PPP2CA,  TSPY1, TSPY2 | 0.0025 |

Enrichment were performed on Biomart-biomarkers, Gene Ontology-biological process, CTD-diseases databases, number of genes enriched are presented with the corrected *p*-value of enrichment after 2000 permutations.
